# Factors Affecting Infestation by *Triatoma infestans* in a Rural Area of the Humid Chaco in Argentina: A Multi-Model Inference Approach

**DOI:** 10.1371/journal.pntd.0001349

**Published:** 2011-10-18

**Authors:** Juan M. Gurevitz, Leonardo A. Ceballos, María Sol Gaspe, Julián A. Alvarado-Otegui, Gustavo F. Enríquez, Uriel Kitron, Ricardo E. Gürtler

**Affiliations:** 1 Laboratory of Eco-Epidemiology, Department of Ecology, Genetics and Evolution, Universidad de Buenos Aires, Buenos Aires, Argentina; 2 Department of Environmental Studies, Emory University, Atlanta, Georgia, United States of America; USAMRIID, United States of America

## Abstract

**Background:**

Transmission of *Trypanosoma cruzi* by *Triatoma infestans* remains a major public health problem in the Gran Chaco ecoregion, where understanding of the determinants of house infestation is limited. We conducted a cross-sectional study to model factors affecting bug presence and abundance at sites within house compounds in a well-defined rural area in the humid Argentine Chaco.

**Methodology/Principal Findings:**

*Triatoma infestans* bugs were found in 45.9% of 327 inhabited house compounds but only in 7.4% of the 2,584 sites inspected systematically on these compounds, even though the last insecticide spraying campaign was conducted 12 years before. Infested sites were significantly aggregated at distances of 0.8–2.5 km. The most frequently infested ecotopes were domiciles, kitchens, storerooms, chicken coops and nests; corrals were rarely infested. Domiciles with mud walls and roofs of thatch or corrugated tarred cardboard were more often infested (32.2%) than domiciles with brick-and-cement walls and corrugated metal-sheet roofs (15.1%). A multi-model inference approach using Akaike's information criterion was applied to assess the relative importance of each variable by running all possible (17,406) models resulting from all combinations of variables. Availability of refuges for bugs, construction with tarred cardboard, and host abundance (humans, dogs, cats, and poultry) per site were positively associated with infestation and abundance, whereas reported insecticide use showed a negative association. Ethnic background (Creole or Toba) adjusted for other factors showed little or no association.

**Conclusions/Significance:**

Promotion and effective implementation of housing improvement (including key peridomestic structures) combined with appropriate insecticide use and host management practices are needed to eliminate infestations. Fewer refuges are likely to result in fewer residual foci after insecticide spraying, and will facilitate community-based vector surveillance. A more integrated perspective that considers simultaneously social, economic and biological processes at local and regional scales is needed to attain effective, sustainable vector and disease control.

## Author Summary

Vector-borne transmission of Chagas disease remains a major public health problem in parts of Latin America. *Triatoma infestans* is the main vector in the countries located in the South American Cone, particularly in the Gran Chaco ecoregion where residual insecticide control has achieved only a moderate, irregular impact. To contribute to improved control strategies, we analyzed the factors associated with the presence and abundance of *T. infestans* in 327 inhabited houses in a well-defined rural area with no recent vector control interventions in the humid Argentine Chaco. Bugs were found mainly in domiciles, kitchens, storerooms, and chicken coops and nests, particularly where adequate refuge and animal hosts (humans, dogs, cats or poultry) were available. Domiciles constructed from mud were the most often infested, but brick-and-cement domiciles, even in good conditions, were also found infested. Availability of refuge and hosts for *T. infestans* are key targets for vector control. Ten-fold variations in domestic infestation observed across neighboring villages, and differences in the relevant factors for *T. infestans* presence with respect to other areas of the Gran Chaco region suggest that host management, building techniques and insecticide use need to be tailored to the local environment, socio-economic characteristics, and climatic conditions.

## Introduction

The transmission of *Trypanosoma cruzi*, the etiologic agent of Chagas disease, by hematophagous triatomine bugs remains a major public health problem in many rural and some periurban communities in Latin America [1]. For decades vector control actions have relied mostly on residual insecticide spraying; implementation has been heterogeneous geographically and impacts on *T. cruzi* transmission variable. In addition to particular regional characteristics and the inherent difficulties encountered in controlling the vectors, *T. cruzi* transmission usually occurs in settings of marginalized human populations [2].

*Triatoma infestans* is not an exception. This species mainly infests poor rural dwellings and the surrounding human-made structures. Although the distribution range of *T. infestans* has been greatly reduced and parasite transmission interrupted in Brazil, Chile and Uruguay [3], residual insecticide spraying has had a moderate, irregular impact in the core of its range where vector elimination has encountered serious obstacles [1], [4], [5]. This area corresponds mainly to the Gran Chaco ecoregion, a 1.1 million km^2^ semiarid plain covering large parts of northern and central Argentina, southeast Bolivia, and central and western Paraguay [6]. The local human population consists mostly of Creoles and several indigenous groups sparsely distributed in rural areas, with scarce access to a very poor infrastructure, including weak health services and institutions under inconsistent provincial and federal policies [7]. These factors modify the effectiveness of vector control operations in an area where *T. infestans* thrives and where environmental conditions make pyrethroid insecticides less effective in some of the most heavily infested peridomestic structures surrounding human dwellings [8], [9]. Peridomestic structures usually play a fundamental role in maintaining abundant triatomine bug populations of various species close to domiciles [8], [10]–[16].

Understanding the factors associated with infestation may help to elucidate how the social dimensions of the problem become materialized; identify possible intervention targets, and predict parasite transmission risks. Broadly speaking, *T. infestans* depends on the availability of three basic biophysical characteristics to establish successful colonies: i) warm-blooded hosts, ii) suitable habitats and climatic conditions, and iii) not being exposed to effective insecticides. Humans, dogs, cats and chickens have repeatedly been identified as important domestic hosts [17]. Suitable bug habitat has often been associated with structural characteristics of houses and peridomestic structures: poor building conditions, wall cracks, mud-and-thatch houses and certain peridomestic structures such as goat or pig corrals and chicken coops [10], [12], [18]–[20]. The application of low-concentration insecticides by householders has been also found negatively associated with infestation [21], [22]. Seldom has the relationship between these various biophysical variables and house infestation been addressed simultaneously in a large number of houses with standardized bug collection methods, detailed information at biologically meaningful units of habitat, and robust analytical methods that reduce biases in variable selection. A multi-model inference approach serves that purpose [23] and has not been applied to Chagas disease vectors.

Despite the wide distribution area of *T. infestans*, variables associated with house infestation by *T. infestans* have been thoroughly assessed in few areas: in the Brazilian Cerrado [10], [24] and in the dry (western) section of the Gran Chaco [8], [21], [22]. However, the ecology of *T. infestans* and parasite transmission patterns may vary between ecoregions depending on climate, environment, human practices and ethnic background. Detailed descriptions of the key elements providing adequate habitats for triatomine bugs have rarely been given. As part of an ongoing multi-site research program on the eco-epidemiology and control of *T. infestans* in the Gran Chaco, the present study aims at modeling the variables associated with the presence and abundance of *T. infestans* in a well-defined rural area in the Argentine Chaco 12 years after the last residual insecticide spraying campaign.

## Materials and Methods

### Study Area and Population

**Figure 1:**
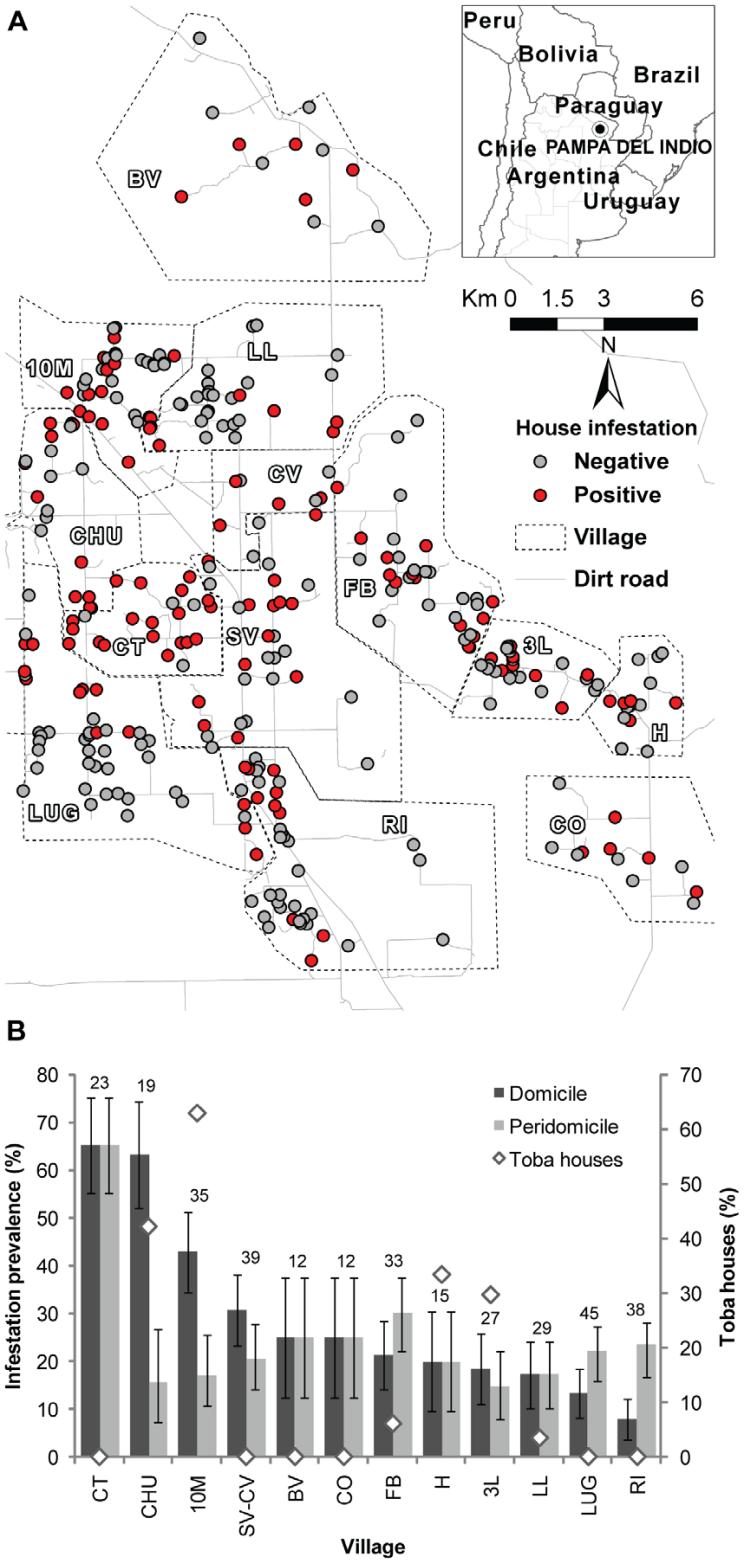
Distribution of house-specific infestation by *T. infestans* according to timed-manual collections in Pampa del Indio. A: Map of the study area with inhabited houses, villages and infestation status. B: domestic and peridomestic prevalence of infestation according to village; diamond symbols denote the percentage of houses inhabited by Toba families in each village; numbers above bars indicate the number of inhabited houses in each village, whiskers indicate ±1 standard deviation. Village acronyms: 10 M, 10 de Mayo; CT, Campo Los Toros; CO, Colonia Ombú; SV-CV, El Salvaje- Los Ciervos; FB, Fortín Brown; H, La Herradura; LL, La Loma; BV, Las Bravas; CHU, Las Chuñas; RI, Santa Rita; LUG, Santos Lugares, and 3L, Tres Lagunas.

The study was conducted in a section (450 km^2^) of the municipality of Pampa del Indio (25°55′S 56°58′W), Province of Chaco, Argentina (Figure 1A, inset), located in the humid (east) Chaco, close to the transition to the dry (west) Chaco. Based on data collected by the Chagas disease control program of Chaco, which indicated high infestation levels, we selected the municipality of Pampa del Indio as this project's location. Based on an exploratory survey throughout the rural area of Pampa del Indio, in which we inspected for triatomine infestation a systematic sample (11%) of the district's houses, we selected a well-defined section with slightly higher infestation than the rest and more than 300 adjacent houses isolated ≥1 km from the nearest villages outside of the selected area.

The study area included 353 houses and several public buildings of 13 neighboring rural villages (Figure 1). The climate is continental, warm, with rain mainly in summer. Annual mean temperature is 22.8°C (mean minimum and maximum, 16.9 and 29.3°C). Annual rainfall historically has been 954 mm, although in 2008 and 2009 a severe drought affected the region. The landscape is flat and comprises mainly a mosaic of patches of crops mixed with native dry forest that has undergone various degrees of degradation, and with occassional water bodies and marshes.

The two main ethnic groups are Creole and Toba. Tobas represent 24% of the 1,187 inhabitants of the study area and occupy 16% of the houses. Creoles are of European descent and usually had a high degree of mixing with indigenous people generations ago. Most Creoles migrated to the area during the last 50–100 years from nearby provinces or from Europe. Tobas –the only indigenous group in the area– were traditionally nomadic or semi-nomadic hunter-gatherers. Following local colonization in the 1920s they began to rely increasingly on agriculture, temporary informal jobs, and state-run welfare programs a few decades ago [25]. Local authorities reported having approximately 5,000 beneficiaries of welfare programs among the ∼14,000 inhabitants in the whole municipality, both Toba and Creoles. Rural residents live mostly on a subsistence economy, and may grow cotton, corn, pumpkins and water-melons or raise livestock (mainly goats, but also cows and occassionally sheep). The nearest hospital is in Pampa del Indio town (∼5,000 inhabitants), 10–45 km away from the study area by dirt roads. The last community-wide insecticide spraying campaign conducted by vector control personnel was carried out in 1996, except for a few houses treated by villagers or hospital staff in 2006, and no specific or systematic educational campaigns regarding Chagas disease or its transmission were performed in the area.

**Figure 2:**
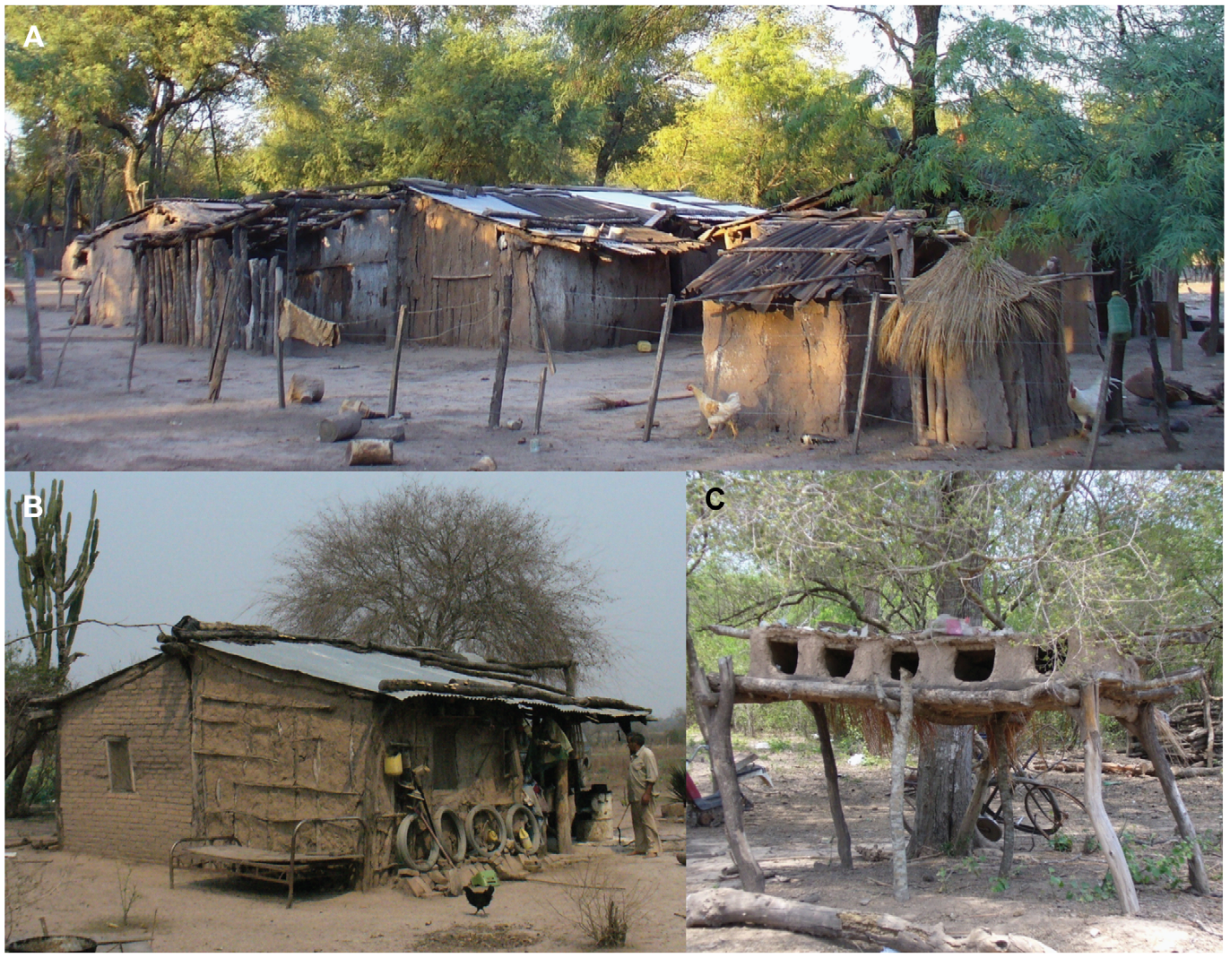
Typical structures of rural Pampa del Indio. A: Domicile and part of peridomicile exhibiting a variety of roof materials (corrugated metal and tarred-cardboard sheets, and thatch). B: A domicile, partly with mud-stick walls, partly with walls made of brick laid on mud, and a corrugated metal roof. C: A mud ‘nidero’ where chickens and other poultry nest.

The study area encompassed 327 inhabited house compounds, including all of its domestic and peridomestic sites. There were 26 uninhabited or abandoned houses and 37 public buildings (including 11 schools, five primary health care centers, and several temples and community centers). A house compound encompassed the domicile and all sites within the peridomestic area (i.e., peridomicile) –usually a latrine, a storeroom, a kitchen, an oven, one or more corrals, and one or more sites for chickens and other poultry (trees, coops, nests) (Figure 2A,B). A site was any individual structure built and/or given a defined use by householders which might provide refuge for bugs. Ecotope (a categorical variable) was defined as a site characterized by some typical structure and use (e.g., domicile, storeroom, chicken coop, etc.). Nests were frequently found within a distinctive structure called ‘nidero’ (from the Spanish for nest, ‘nido’, Figure 2C), consisting of an elevated shelf made of wood or sometimes bricks where chickens and, occasionally, turkeys or ducks nested. Most domiciles were mud-and-thatch huts with corrugated metal or tarred-cardboard roof.

### Household Survey

We conducted a cross-sectional survey of all houses in the study area accompanied by local personnel (health-care agent, vector control personnel) to explain to householders the goals of the research in a clear and simple language and to request access to their premises in September 4–15 and October 24–November 17, 2007, following protocol approved by IRB No. 00001678 (NIH registered). The full name of the head of each household and numbers of resident people aged 0–4 and 5–14 years old were recorded at each house. A sketch map of the spatial setting of the different structures in the house compound was drawn. Each structure was given an individual code according to its use to unequivocally identify it in follow-up surveys and associate it with the collected triatomines. The location of each site was georeferenced with a GPS receiver (Trimble GeoXM or Garmin Legend). After seeking householders' permission, an aluminum numbered plate was placed by the main entrance of the domicile and in public buildings. A labeled self-sealing plastic bag was provided to each household to keep any triatomine bug they may capture in domestic or peridomestic sites after insecticide spraying. Householders were asked to keep the bugs until our next visit and were instructed on how to collect the bugs without incurring a contamination risk.

Building materials used in roofs and walls, presence of wall plaster, condition of wall surface, plaster material, ecotope and physical characteristics of each site were recorded. The availability of suitable refuges for *T. infestans* was assessed visually by an experienced bug collector of the research group, and rated from 1 (no refuge at all) to 5 (abundant refuges). The head of each household (or some other adult person) was asked the number and type of domestic animal they owned, their resting sites, and domestic use of insecticides (type, frequency, mode and date of last application). Data on reported insecticide use, ecotope, construction materials, refuge availability, numbers of people and hosts and their resting places were collected in April, August and December 2008. Because animal host numbers varied seasonally, data collected in December 2008 were used to describe host numbers for the initial survey conducted in October 2007. This may introduce some inaccuracies, particularly in sites seldom used by one or very few individual hosts, such as some chickens or other poultry nesting in a domicile or storeroom, as this could vary from year to year. For all other variables with no seasonal variations, data collected on April 2008 were used; when values were missing, data collected in the following survey were used.

To evaluate whether the visual assessment of refuge availability of a site varied between different observers, after a preliminary standardization of criteria in two houses six similarly experienced bug collectors familiar with the study area independently scored refuge availability in 145 sites from 45 houses. These bug collectors were grouped in three different teams that scored the sites inspected for bugs during one day of survey.

### Entomological Survey

Simultaneously with the household survey, all sites within each house compound were searched for triatomine bugs by timed manual collections conducted by two skilled bug collectors from the national or provincial vector control programs using 0.2% tetramethrin (Espacial, Argentina) as a flushing-out agent. Domiciles were inspected by one person for 20 min whereas each peridomestic site was searched by one person for 15 min. In practice, most searches lasted less because the site was completely inspected before the stipulated time. Therefore, the duration of searches in domiciles and in other ecotopes was approximately similar and therefore, the search effort was rounded to 0.25 person-hour per site. Public buildings were inspected in the same fashion. In several houses bugs were also collected after the stipulated search time (after-manual collections), during insecticide applications, or by householders a few days after timed manual collections or insecticide spraying. These additional bug collections were used solely as a qualitative measure of infestation. Householders' bug collections were only considered when accompanied with date and place of collection. Immediately after the entomological survey, all sites from every house were sprayed with suspension concentrate deltamethrin (K-Othrin, Bayer) at standard dose (25 mg/m^2^) by vector control personnel. The collected triatomine bugs were transported to the field laboratory in plastic bags labeled with bug collection site, identified taxonomically and counted according to species, stage or sex [26].

### Data analysis

All results are reported solely for inhabited houses unless otherwise noted; uninhabited houses and public buildings were not found to be infested. Prevalence of infestation and colonization by *T. infestans* was calculated both for site or house compound levels. A site was considered infested when at least one live *T. infestans* nymph or adult was found by timed-manual collections. If at least a live nymph of *T. infestans* was found, the site was additionally considered colonized. Bug abundance was computed as the number of live *T. infestans* collected in a specific site by timed-manual collection per unit effort. The prevalence of site-specific infestation (or colonization) was calculated as the total number of infested (or colonized) sites divided by the total number of sites inspected for infestation. House-level infestation (or colonization) prevalence was calculated as the total number of infested (or colonized) house compounds divided by the total number of house compounds inspected for infestation.

The relationship between study factors and site-specific infestation or bug abundance per unit effort was assessed by means of logistic and negative binomial regression models, respectively. Negative binomial regression was chosen instead of Poisson regression because bug abundance per site was overdispersed. This was reflected in extremely larger values of Akaike's information criterion (AIC) for Poisson models than for negative binomial models (data not shown). Analysis was restricted to the most frequently infested ecotopes (i.e., kitchens or storerooms, domiciles, chicken coops, and ‘nideros’) because maximum likelihood procedures did not converge when other rarely infested ecotopes were included in negative binomial models. Regression analyses were performed for site level (i.e., each individual observation in the regressions represented a site).

Two separate sets of analyses were performed according to the typical characteristics of ecotopes to address the fact that the physical structure of a site affects bug detectability and therefore, comparability of results. One set of analyses included ecotopes typically made of proper walls and roofs (i.e., domiciles, kitchens and storerooms); the second set included small (peridomestic) ecotopes used by chickens (i.e., chicken coops and ‘nideros’). In both cases the response variables were site-specific infestation for logistic regressions and bug abundance per unit effort for negative binomial regressions. For domiciles, kitchens and storerooms, predictors included in the model were ethnic group, householders' reports of insecticide use, ecotope (with two categories, taking domiciles as the reference category), building materials (each material was taken as a separate variable since several of them could be present within the same site), refuge availability, numbers of people, dogs or cats, poultry (mostly chickens), and fledglings. For chicken coops and ‘nideros’ the same predictors were included, except ethnic group (due to the extremely few such sites among Tobas); householders' insecticide use (virtually restricted to domiciles, kitchens and storerooms), and numbers of people, dogs and cats (not reported to rest in chicken coops or ‘nideros’).

Pair-wise correlation coefficients between predictors were low (−0.2<r<0.2) for all pairs of variables except for: mud and refuge availability (r = 0.38), mud and thatch (r = 0.39), metal and brick (r = 0.32), domiciles and wood (r = −0.27); all these correlation coefficients were statistically significant (P<0.05). However, due to the possibility of linear dependencies involving more than two variables (i.e., multicollinearity) and its eventual detrimental effects on parameter estimation, the condition numbers of the predictors matrix were calculated [27]. The largest condition number was 8, indicating weak dependencies among variables that should not imply estimation problems [27].

A multi-model inference approach based on AIC was used to assess the relative importance (RI) of each variable according to Burnham and Anderson [23]. This procedure provides a quantitative ranking of the relative contribution of each variable to model fitting given the variables and models considered, while helping to reduce overfitting. All models considering every possible combination of the study variables were run: 14 variables gave 16,383 models for domiciles, kitchens and storerooms, and 10 variables gave 1,023 models for chicken coops and ‘nideros’. We considered all variables in equal terms since there was virtually no evidence on their relative importance when considered simultaneously. Therefore, the models considered were not alternative sets that evolved over time. The relative likelihood (i.e., Akaike weights) of each model was calculated as the quotient of the log-likelihood of the particular model divided by the total sum of the log-likelihoods of all considered models. The RI of each variable was calculated as the sum of the Akaike weights over all models in the set where the respective variable was present. The maximum value RI can take is 1, representing maximum importance relative to the set of variables considered, whereas RI = 0 represents no importance at all relative to the set of variables considered. Parameter estimates for each variable resulted from averaging the parameter value in each model where the variable was present weighted by the Akaike weight of the respective model. The overall quality of the fitted logistic regression models was assessed by means of the Hosmer and Lemeshow goodness-of-fit test using the averaged coefficients and grouping the data in 10 equal-sized groups; the area under the receiver operating characteristic (ROC) curve; sensitivity and specificity, using as cutoff values the observed prevalence of infestation for each data set (i.e., 17.7% for domiciles, kitchens and storerooms; 14.8% for chicken coops and ‘nideros’). Analyses and calculations were performed in R 2.7.0 [28]; the scripts with the commands for running the models and the respective calculations were prepared using a Visual Basic macro on Microsoft Excel (Text S1 and Script S1); model fitting was assessed in Stata 9.0 [29].

The spatial distribution of infested sites was assessed using the random labeling method with the pair-correlation function implemented in Programita [30]. The pair-correlation function *g*_12_(*r*) [31] evaluates if the number of points of pattern 2 within a ring of radius *r* and a given width, centered at each point of pattern 1, corresponds on average to a random process (i.e., a homogeneous Poisson process; *g*_12_(*r*) = 1), aggregation of 2 around 1 (*g*_12_(*r*)>1), or regularity of 2 with respect to 1 (*g*_12_(*r*)<1). The random labeling method assesses the spatial distribution of points belonging to a given pattern –relative to other points belonging to either that or other pattern– taking into account the spatial distribution of all points. Several site-specific characteristics affect the probability of being infested, regardless of the vicinity of other infested sites. Therefore, in the present case the null model for reassigning the membership to either pattern (and thus generating the expected distribution) took into account the probability of being infested according to the logistic regression model with all the variables weighted according to their RI. The spatial analysis only included sites pertaining to the four ecotopes most frequently infested (i.e., domiciles, kitchens and storerooms, chicken coops and ‘nideros’). The grid size for analysis was 100 m; ring width, 400 m; maximum radius, 5 km; 999 simulations were performed, and the upper and lower 25^th^ simulations were used as a 95% confidence envelope. A goodness-of-fit test [30] was used to evaluate the overall fit of the observed pattern to the expected distribution.

To analyze the consistency among different observers of the visual estimation of refuge availability, the kappa index of agreement was calculated using Stata 9.0 [29]. This measure of agreement reaches the value of 1 when there is complete agreement and zero if observed agreement is equal to random agreement. Values>0.60 may be considered substantial to perfect agreement; values<0.40 represent poor agreement beyond chance, whereas values between 0.40 and 0.60 may be interpreted as moderate agreement beyond chance [32].

## Results

A total of 2,584 domestic or peridomestic sites was georeferenced, mapped and inspected for infestation. *T. infestans* was found by timed manual collections in 39.8% of the 327 inhabited house compounds and in only 7.4% of all sites inspected; bug colonies occurred in 30.8% of inhabited house compounds. Infestation with *T. infestans* was detected in 25.9% of domiciles and in 26.2% of peridomiciles. *Triatoma sordida* was found in 18.3% of houses, in 1.2% of domiciles (only adults) and in 17.6% of peridomiciles. A total of 2,062 *T. infestans* and 331 *T. sordida* were collected by timed manual collections. House compound infestation with *T. infestans* reached 45.9% when results from all collection methods were pooled together.

### Ecotopes: infestation and building characteristics

**Figure 3:**
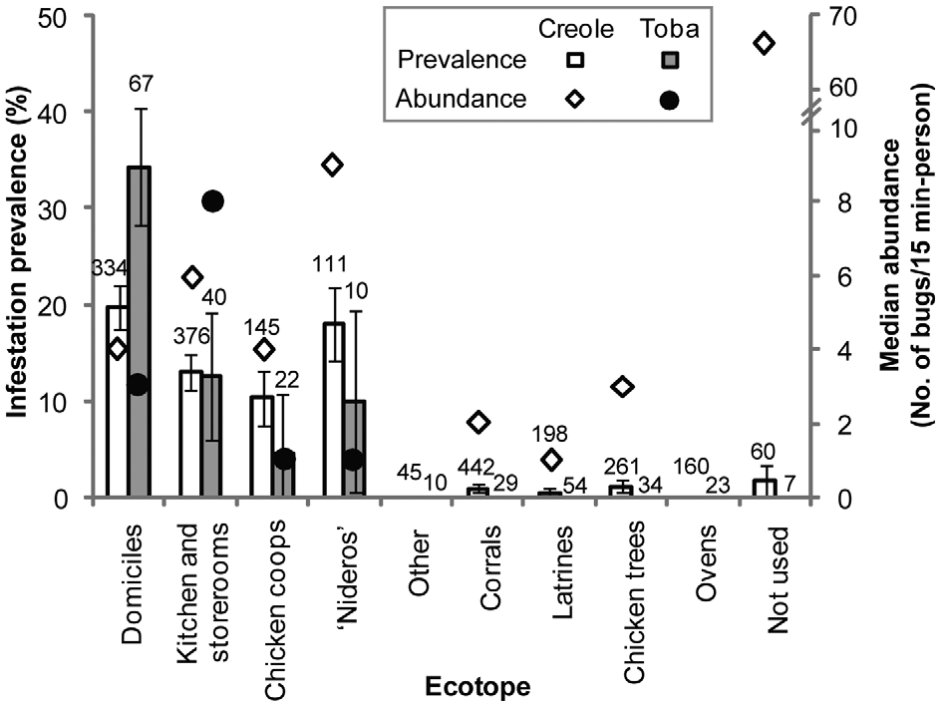
Prevalence of infestation and median abundance of *T. infestans* according to ecotope and ethnic group. Data corresponds to timed-manual collections. Numbers above bars indicate the number of sites examined for infestation within each category. Whiskers indicate ±1 standard deviation.

**Figure 4:**
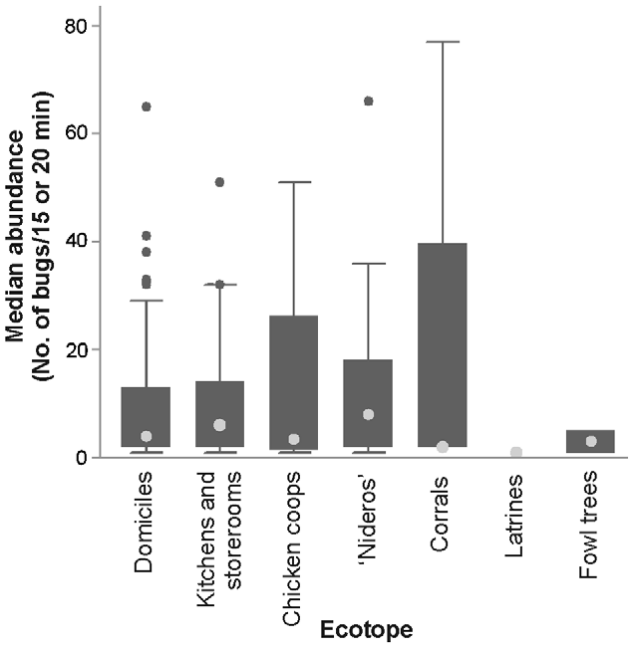
Abundance of *T. infestans* per site in infested sites according to their ecotope. Bug abundance determined by timed-manual collections. Light gray symbols indicate median abundance; boxes, interquartile range; whiskers, values adjacent to interquartile range; dark symbols, outside values.

The most frequently infested ecotopes were domiciles (25.8%), kitchens or storerooms (15.4%), chicken coops (13.2%), and ‘nideros’ (18.2%), whereas <1.0% of corrals (including goat, pig and cow corrals), latrines, trees used by chickens and other ecotopes (ovens, chapels, abandoned cars, piles, etc.) were infested (Figure 3). The relative abundance of *T. infestans* tended to peak in ‘nideros’ followed by kitchens or storerooms, and was highly variable within and between ecotopes (Figure 4). Among frequently infested ecotopes, infestation in chicken coops was the most variable. Goat corrals and other types of corrals were rarely infested. In corrals, the mean bug abundance was widely variable but this was due to the very large number of bugs (80) collected at one of the four infested sites.

Most domiciles were built, partly or wholly, with mud (55.2%) and corrugated metal-sheet roofs (66.2%) (Table 1). Of domiciles with metal-sheet roofs, a substantial fraction had part of the roof made of thatch (14%) or corrugated tarred-cardboard sheets (7%) and ceilings made of wood (14%) or cane (2%), all of which provided suitable refuges for bugs. Mud, mixed with straw, was mainly applied onto a wooden frame for building walls (Figure S1A,G), and less frequently it was used for laying bricks instead of cement (Figure S1F). Deteriorated mud walls tended to have big cracks as the mud separated from the logs of the wooden frame of walls (Figure S1B); minor cracks were frequently observed in the mud plaster. In many heavily infested sites, no fecal streaks of triatomine bugs were found on wall surfaces but such streaks were abundant within cracks along the wooden frame of walls. Metal sheets were mostly used for roofs and, in a few cases, walls. Domiciles made solely of brick-and-cement walls and with metal-sheet roofs (comprising 36.4% of all domiciles) were also found infested but had substantially lower infestation (15.1%), colonization (6.2%) and refuge availability index (mean ± SD = 2.9±1.0) than domiciles with mud walls and roofs made of thatch or sheets of corrugated tarred-cardboard (32.2%, 21.6% and 3.8±0.9, respectively).

**Table 1:** Use of building materials and refuge availability (mean ± standard deviation, SD) according to ecotope.

		% of sites built with							Refuge availability (mean ± SD)
Ecotope	No. of sites	Mud	Wood^a^	Thatch^b^	Cardboard^b^	Metal sheets^b^	Brick	Others	
Domiciles	391	55.2	19.7	24.8	20.7	66.2	44.0	33.8	3.4±1.0
Kitchens or storerooms	379	62.3	37.2	28.8	25.9	45.9	18.2	14.0	3.0±1.0
Chicken coops	121	16.5	80.2	6.6	16.5	21.5	12.4	26.4	2.5±1.1
‘Nideros’	109	28.4	70.6	8.3	14.7	24.8	48.6	22.9	3.3±1.0
Other*	41	19.5	24.4	14.6	9.8	48.8	36.6	34.1	1.9±0.9
Corrals	436	0.5	90.6	2.1	4.8	8.5	0.5	31.2	2.0±0.7
Latrines	239	34.3	23.4	7.9	20.9	47.7	56.5	43.9	2.3±1.0
Ovens	161	46.0	41.0	0.6	0.6	2.5	88.8	39.8	2.2±1.0
Not used	46	19.6	28.3	8.7	19.6	32.6	21.7	23.9	2.3±0.8

*‘Other’ includes mainly plastic items and clothes.

a A site was considered to have wood as a building material when an important part of the site had wood uncovered. Wood is usually present in the structure on which mud is laid, but in those cases it was not considered a building material.

b In domiciles, kitchens and storerooms, the material in question was mostly in the roof. In the case of cardboard, as tarred-cardboard sheets.

**Figure 5:**
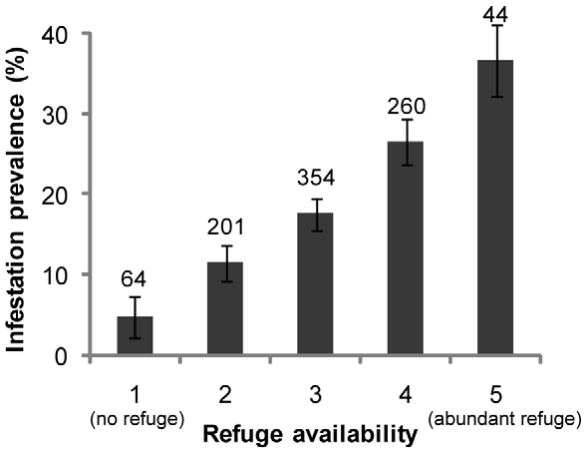
Prevalence of infestation with *T. infestans* according to refuge availability among the main infested ecotopes. Numbers above points indicate the number of sites inspected in each category. Whiskers indicate ±1 standard deviation.

Kitchens and storerooms had similar construction features as domiciles, though brick walls were less frequent (Table 1). These ecotopes frequently exhibited a remarkable patchy distribution of building materials (Figure 2A,B), reflecting the changing access to building materials over time. Chicken coops were largely made of wood (80.2%); <17% of chicken coops was built with mud, brick, cardboard (either as tarred-cardboard sheets or as cardboard boxes) or thatch. ‘Nideros’ were similarly made as chicken coops though mud and bricks were more frequent and were usually used for building the small walls that separated one nest from the next (Figure 2C). Corrals were largely made of wood. Goat corrals were fenced with logs, boards or wire, and rarely had a roofed part for kids to shelter; when they did, the roof was typically made with corrugated metal sheets or wood boards and, more rarely, with tarred-cardboard sheets or thatch. Site-specific infestation by *T. infestans* and the index of refuge availability showed a close, positive association (Figure 5). The most frequently infested ecotopes also had higher refuge availability indices (Table 1, last column). Inter-observer agreement on the refuge availability index ranged between moderate and substantial and was significantly different from agreement by chance alone (kappa index = 0.56, z = 11.67, P<0.0001). Ratings between observers coincided in 68% of the rated sites, and differed by only one unit in 28% of the sites.

### Host abundance and distribution

Dogs and cats were the most frequent domestic animal hosts resting in domiciles, kitchens or storerooms as reported by householders (Table S1). Dogs were reported to sleep indoors rarely, though they usually rested against the external walls of domiciles or in verandas. Chickens and other poultry were reported to rest at night mainly on trees and secondarily in chicken coops. Fledglings occurred in all peridomestic ecotopes. Corrals, latrines and ovens were rarely used by chickens, dogs or cats. Goats, sheep, pigs and cows occupied almost exclusively their respective corrals (having few refuges) where *T. infestans* was hardly ever found.

### Ethnic group and infestation

Toba households were larger and younger than Creole households, had more dogs, and substantially fewer chickens, goats, pigs, cows and equines than Creoles (Table 2). On average, Toba households had fewer sites per house compound (5.8) than Creole's (7.9), mainly due to fewer kitchens or storerooms, corrals, and peridomestic structures housing chickens (Table S2). The prevalence of house infestation with *T. infestans* (as determined by any collection method) was 58.8% for Tobas and 43.5% for Creoles (χ^2^ = 4.1, d.f. = 1, P = 0.04). Infestation was higher in Tobas' domiciles, kitchens or storerooms, and lower in Tobas' chicken coops and ‘nideros’ than in those owned by Creoles (Figure 3). The median abundance of *T. infestans* was similar between ethnic groups in domiciles, much higher in chicken coops and ‘nideros’ among Creoles, and slightly higher among Tobas in kitchens or storerooms (due to two sites with >12 bugs collected).

**Table 2:** Domestic hosts of each type per inhabited house compound according to resident ethnic group.

	Mean no. per house ± standard deviation (% households with host)											
Host	Creole				Toba				Total			
Total people	3.6	±	2.4	(97.4)^a^	5.5	±	3.1	(98.0)^a^	3.9	±	2.6	(97.5)^a^
0–4 yr old	0.4	±	0.9	(28.6)	1.0	±	1.0	(56.8)	0.5	±	0.9	(33.0)
5–14 yr old	0.8	±	1.2	(39.8)	1.4	±	1.4	(62.7)	0.9	±	1.3	(43.4)
Dogs	2.6	±	2.1	(80.4)	3.2	±	2.2	(86.2)	2.7	±	2.1	(81.3)
Cats	0.7	±	1.1	(45.6)	0.7	±	1.2	(39.2)	0.7	±	1.1	(44.6)
Poultry	33.8	±	35.1	(80.7)	23.3	±	31.8	(84.3)	32.1	±	34.8	(81.3)
Goats	14.9	±	31.0	(42.1)	3.8	±	9.0	(29.0)	13.0	±	28.8	(39.8)
Pigs	4.2	±	6.9	(58.6)	1.0	±	2.6	(20.0)	3.7	±	6.5	(52.0)
Cows	29.4	±	78.7	(45.4)	4.8	±	19.4	(27.2)	25.2	±	72.7	(42.3)
Equines	2.8	±	6.1	(35.7)	1.8	±	3.8	(36.3)	2.7	±	5.8	(35.8)

a Some houses had temporally no resident people only.

Reported use of some kind of insecticide during the previous year was higher among Creoles (64%) than Tobas (41%). Among families reporting insecticide use, 81.3% of Tobas and 77.5% of Creoles applied low-concentration pyrethroid sprays more than once a month, and 6.3% and 5.6% applied them between three and 12 times a year, respectively. Only 6.3% of Tobas and 8.1% of Creoles reported applying high-concentration pyrethroid or carbamate insecticides with manual compression sprayers (used for agriculture) once or twice a month, whereas 6.3% and 8.7% reported applications once or twice a year, respectively. Among households reporting insecticide use, infestation prevalence was lower for Creoles than Tobas in domiciles (19.7% vs. 28.3%), kitchens or storerooms (11.0% vs. 18.4%), and chicken coops (9.7% vs. 14.3%), respectively. Domestic infestation prevalence varied ten-fold across villages, whereas peridomestic infestation tended to be more similar between most villages (Figure 1B). Of the two villages with very high domestic infestations (>60%), one was inhabited only by Creole families whereas in the other village half of the households were Toba.

### Multivariate analyses

The multivariate analyses of factors associated with site-specific infestation and abundance of *T. infestans* in the most frequently infested ecotopes are shown in Table 3. In domiciles, kitchens and storerooms, refuge availability showed maximum RI (1.00) for infestation and bug abundance. Insecticide use (RI = 0.99), numbers of people and dogs or cats (RI = 0.95–0.96), and having a tarred-cardboard roof (RI = 0.86) all had higher RI regarding infestation than for bug abundance. Insecticide use was the only factor that had a negative association with infestation or bug abundance. Ethnic group evidenced a low RI regarding infestation and a moderately high RI in relation to bug abundance. In chicken coops and ‘nideros’, the numbers of chickens were important for both infestation and bug abundance whereas cardboard, thatch and mud were more important in relation to infestation than bug abundance, all with large positive effects. Refuge availability showed no clear importance in chicken coops and ‘nideros’.

**Table 3:** Relative importance and effects of variables in relation to site infestation and abundance of *T. infestans*.

	Domiciles, kitchens and storerooms							Chicken coops and ‘nideros’						
		Infestation			Bug abundance				Infestation			Bug abundance		
Variable	% infested (No. of sites inspected)	RI	OR	S.E.	RI	Coef.	S.E.	% infested (No. of sites inspected)	RI	OR	S.E.	RI	Coef.	S.E.
Refuge availability	17.7 (770)	1.00	1.59	0.53	1.00	0.72	0.38	14.8 (230)	0.50	1.16	0.45	0.68	0.61	0.57
Insecticide use		0.99			0.76									
No	25.2 (262)		1.00			0.00								
Yes	13.8 (508)		0.23			−0.52	0.57							
No. of people	22.8 (382)	0.95	1.16	0.23	0.44	0.04	0.14							
No. of dogs and cats	21.9 (183)	0.96	1.16	0.24	0.44	0.03	0.15							
No. of chickens	23.6 (148)	0.27	1.00	0.26	0.31	−0.02	0.09	15.1 (86)	0.95	1.03	0.09	0.95	0.04	0.02
No. of fledglings	16.0 (75)	0.28	1.00	0.06	0.28	0.00	0.04	16.7 (30)	0.30	1.00	0.04	0.50	−0.03	0.04
Cardboard	28.5 (179)	0.86	1.68	0.04	0.77	0.63	0.64	27.8 (36)	0.85	2.86	1.84	0.65	1.08	1.12
Mud	23.2 (452)	0.43	1.15	0.85	0.41	0.17	0.38	27.5 (51)	0.81	2.34	0.90	0.70	1.25	1.14
Thatch	22.3 (206)	0.33	1.06	0.38	0.41	0.17	0.37	41.2 (17)	0.83	3.25	1.21	0.63	1.13	1.24
Brick	10.0 (241)	0.60	0.74	0.22	0.54	−0.38	0.56	20.6 (68)	0.61	1.60	0.90	0.55	0.70	0.87
Metal sheets	13.6 (433)	0.61	0.77	0.35	0.54	−0.31	0.49	17.0 (53)	0.38	1.21	0.59	0.29	−0.03	0.26
Wood	19.7 (218)	0.27	1.01	0.32	0.31	0.06	0.23	14.9 (174)	0.27	1.02	0.23	0.57	−0.67	0.81
Ecotope		0.42		0.14	0.66				0.36			0.39		
Domiciles/Chicken coops	22.5 (391)		1.00			0.00		12.4 (121)		1.00			0.00	
Kitchens or storerooms/‘Nideros’	12.7 (379)		0.29	0.29		−0.44	0.57	17.4 (109)		1.16	0.66		0.28	0.56
Ethnic group		0.34			0.65									
Creole	16.4 (665)		1.00			0.00								
Toba	25.7 (105)		0.22	0.22		−0.57	0.68							

Two separate analyses are displayed according to type of ecotope. Infestation data by timed-manual collections with a dislodging spray were modeled using logistic regressions; timed-manual bug abundance was modeled using negative binomial regression. The relative importance (RI) of variables was assessed by multi-model inference based on Akaike's information criterion. OR: odds ratio; Coef.: coefficient; S.E.: standard error.

The averaged logistic model for infestation in domiciles, kitchens and storerooms had a marginally significant fit (Hosmer-Lemeshow χ^2^ = 14.9, 8 d.f., P = 0.06). The area under the ROC curve was 0.742; sensitivity was poor (49%) and specificity higher (82%), with 76% of observations correctly classified. The averaged model for chicken coops and ‘nideros’ had a good fit (Hosmer-Lemeshow χ^2^ = 4.5, 8 d.f., P = 0.81); the area under the ROC curve was 0.759; sensitivity of 65% and specificity of 71%, with 70% of observations correctly classified.

The averaged negative binomial models in both groups of ecotopes exhibited significant correlations between observed and predicted bug abundances (for domiciles, kitchens and storerooms, r = 0.23; for chicken coops and ‘nideros’, r = 0.61). However, these models considerably underestimated the frequency of zero bugs per site and overestimated higher bug catches (data not shown).

### Spatial analysis

**Figure 6:**
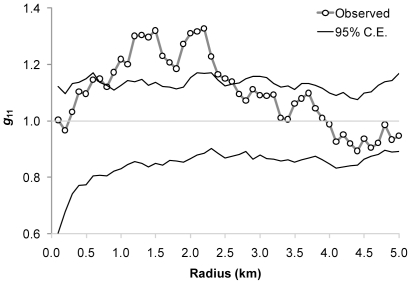
Spatial analysis of site-specific infestation by *T. infestans* among the main infested ecotopes. Only domiciles, kitchens, storerooms, chicken coops, and ‘nideros’ are considered. C.E.: confidence envelope according to the null model (random labeling) under a homogeneous Poisson process.

The spatial distribution of sites infested with *T. infestans* (as determined by any collection method) was assessed only among the most frequently infested ecotopes. Infested sites were significantly aggregated at distances between 0.8–2.5 km (Figure 6). This aggregation remained significant even after taking into account the probability of finding each infested site according to a logistic regression model that included insecticide use, ecotope, refuge availability, building materials, and number and type of animal hosts.

## Discussion

Through a multi-model inference approach we showed that availability of appropriate refuges for *T. infestans* bugs, use of cardboard as a building material and domestic host abundance (humans, dogs, cats, and chickens) were strongly and positively associated with bug infestation and abundance, whereas the association with reported insecticide use by householders was negative in a high-risk rural area in the Argentine Chaco. It is noteworthy that ethnic background, when adjusted for other factors, had little or no association with infestation.

Refuge availability was a key variable for describing the site-specific presence and abundance of *T. infestans* in domiciles, kitchens and storerooms both in univariate and multivariate analysis, having higher relative importance than building materials or host abundance. Refuge availability was also moderately important for bug abundance in chicken coops and ‘nideros’. Refuge availability was demonstrated experimentally to be essential for the development of *T. infestans* colonies [20], but most field studies addressed its effects on domestic infestation only indirectly by assessing the types of wall and roof materials, and only a few considered the degree of wall cracking explicitly [21], [22], [33]. However, wall cracks represent only a fraction of the possible refuges in sites with proper walls, such as domiciles, kitchens and storerooms. The refuge index probably captured part of the effects of mud as a building material as both factors were moderately correlated (r = 0.38). Use of mud has been consistently found to be an important factor associated with infestation throughout the Gran Chaco and in other regions [34] because it frequently cracks (Figure S1A) and therefore provides adequate refuges for triatomine bugs, unless well maintained as in Figure S1G [26]. However, in our multiple regression analysis the inclusion of refuge availability, mud and thatch were moderately associated only with infestation in chicken coops and ‘nideros’, and not at all with infestation in domiciles, kitchens or storerooms. Even though estimates of refuge availability may appear to be a subjective and imprecise measure, concordance among several experienced bug collectors familiar with the study area was moderate to substantial. Therefore, refuge availability may be used as an indicator of habitat suitability for *T. infestans*.

A striking demonstration of the effects of refuge availability on infestation with *T. infestans* is provided by local goat corrals. Because of the type of construction, most goat corrals in Pampa del Indio had very few or no refuges for bugs and were rarely infested (Figure S1I,J). In contrast, goat corrals in the dry Chaco were typically constructed with fences made of piled thorny shrubs and a thatched or earth-made roofed area for shelter, and were very often heavily infested before and soon after residual insecticide spraying [8], [35].

Cardboard material (either as boxes or as tarred-cardboard sheets) used in ‘nideros’, chicken coops and corrals was associated positively and directly with infestation; it provides excellent refuges for *T. infestans* and is also easy to inspect for bugs. In contrast, in domiciles, kitchens and storerooms (where cardboard as a building material is only present in roofs, Figure 2A) no bugs were collected from cardboard material itself. This is hardly surprising because roofs are more difficult to search for bugs, especially high roofs. Corrugated cardboard-made roofs may also be linked to infestation indirectly since they characterize households with fewer resources. Rural villagers reported that cardboard sheets were much cheaper than metal sheets and easier to get than appropriate thatch for roofing (*Spartina densiflora*, locally known as ‘espartillo’). Cardboard use exemplifies the variable and complex relationships that an apparently simple building material may have with several other relevant factors.

Insecticide use by householders had high RI for infestation in domiciles, kitchens and storerooms. The frequent domestic use of insecticides in Pampa del Indio may explain, at least in part, why the observed prevalence of house infestation with *T. infestans* was much lower than expected, considering that the most recent residual spraying with insecticides conducted by the vector control program occurred 12 years earlier, and other areas in the dry Chaco experience fast reinfestation after insecticide spraying campaigns [4], [8], [9], [36]. Insecticide use may also reflect to some extent the economic status of householders and/or their concern for and response to the presence of bugs and other domestic pests [22]. Additionally, our study area exhibited a relatively high proportion of brick-and-cement houses with corrugated metal roofs (36.4%). Although such construction materials do not fully impede house invasion and colonization by triatomine bugs, they probably present much more of an obstacle than mud-and-thatch huts. Local residents frequently recalled that *T. infestans* had been much more abundant a few decades ago and attributed the decline to earlier vector control campaigns (traditionally lacking an educational component and not followed by a vector surveillance and response system), domestic insecticide use, declining use of thatch for roofs and mud for walls, and deforestation. Villagers usually considered the surrounding forest as the main source of *T. infestans*. Although sylvatic foci of *T. infestans* have not been detected in the study area so far (Alvarado-Otegui et al., unpublished data), they have been reported elsewhere in the Gran Chaco [37]–[39]. Taken together, householders' reports and the mixture of building materials recorded (Figure 2B) indicate a slow, gradual trend toward improvement of rural housing in this area of Chaco.

The abundance of certain host species at site level was an important factor in describing variations in infestation and bug abundance. Dogs, cats and people were associated positively with variations in infestation in domiciles, kitchens and storerooms as in the dry Chaco [21], [22] and elsewhere for other triatomine species [40]–[42]. The number of domestic chickens (and some other poultry) was highly relevant for infestation in chicken coops and ‘nideros’, as it was elsewhere in the Argentine dry Chaco [17], [22], [43]. However, such expected effects were not detected in domiciles, kitchens and storerooms, probably due to the relative imprecision of householders' reports on host presence at a given site combined with the lower frequency of chickens nesting in domiciles in comparison to previous study areas.

Differences in health indicators between ethnic groups abound in the literature [44], but we were unable to find any comparisons of levels of house infestation with triatomine bugs between Creole and indigenous populations within the same area. Our results did show that Toba households, which had several demographic differences and used insecticides less frequently than Creole households, had higher infestation in domiciles, kitchens and storerooms. These differences are consistent with the much higher seroprevalence of *T. cruzi* among Tobas (69.9%) compared with Creoles (40.4%) residing close to our study area [45], whereas 54% of Tobas were seropositive in an unspecified section of Pampa del Indio [46]. However, in our multiple regression analysis, when ethnic group was considered simultaneously with other relevant variables, its relationship with infestation was not important. Ethnic group could be relevant in understanding the distribution of *T. infestans* insofar as it is a surrogate of factors directly linked to infestation (e.g., insecticide use). An excessive focus on ethnic background, construed mainly as cultural differences, may obscure other substantial variations within and between Tobas and Creoles. These differences may be related to resources, insecticide use, types and numbers of peridomestic structures and demographic features. Indeed, we documented large differences in house infestation between and within study villages, even among those only having Creole households. Whether differences in infestation between and within Tobas and Creoles reflect average differences in social and economic position between and within groups [47] rather than cultural or behavioral differences that modify infestation merits further inquiry.

A major finding of our study is the marked heterogeneity in the distribution of infestation, housing construction patterns (Figure S1) and several risk factors within an apparently homogeneous rural area. Domestic infestation varied ten-fold among villages, and there was a significant spatial aggregation of infestation. While some houses were heavily infested, 54% of houses were apparently free of *T. infestans* despite the absence of recent vector control actions. Moreover, the presence of the vector species was widespread throughout the study area. Both of these findings imply that *T. infestans* already had sufficient time and opportunities to reach the most suitable sites for colonization [4]. The significant spatial aggregation of infested sites within a range of 0.8–2.5 km suggests the contribution of factors related to infestation that were not explicitly included in our models. Such putative factors that may contribute or underlie the aggregated occurrence of *T. infestans* are the economic status of householders (e.g., by affecting housing quality or maintenance, and access to insecticides), extended family clusters (which usually live in nearby houses and probably share similar housing characteristics, domestic and productive practices), cultural groupings (such as Tobas, who tend to live in clusters) and productivity (as it strongly affects economic status), all of which may be spatially clustered and eventually interrelated. This underscores the need for identification of relevant socioeconomic variables for an improved understanding of system structure and dynamics.

We found substantial differences regarding host availability and local characteristics of adequate habitats for triatomine bugs in comparison to other study areas in the Argentine dry Chaco [9], [22], [35]. The local use and relevance for infestation of tarred-cardboard and metal sheets as building materials contrast with its rarity elsewhere. ‘Nideros’, highly infested and widespread in Pampa del Indio (Figure 2C), are virtually absent in the dry Chaco. Goat corrals were rarely infested in Pampa del Indio and often heavily infested in the dry Chaco for reasons explained above. Domiciles in Pampa del Indio are roofed mostly with corrugated metal or tarred-cardboard sheets and occasionally thatch (Figure S1C,G), verandas are rare and small (Figure 2B), and most bugs are collected from walls and stored goods. In contrast, in the dry Chaco most domiciles have thatch-and-earth roofs (where *T. infestans* is very common) and large verandas where people sleep during the hot season [21], [22], [48], [49]. These contrasting patterns likely derive from differences in climate (i.e., earth-and-thatch roofs deteriorate fast with more rainfall in the humid Chaco); domestic animal management practices (influenced by traditions, local needs and possibilities and other factors); local political practices (corrugated metal and cardboard sheets were frequently provided pro bonus by political candidates in Pampa del Indio), and more intense activities of charity organizations. Taken together with the rich environmental, cultural and climatic diversity within the Gran Chaco, our findings emphasize the need of considering local specificities and processes (including householders' practices) as these may vary widely within the same region and modify the likelihood for house infestation in unforeseen ways.

Cross-sectional, observational field studies such as ours have inherent limitations and strengths. Timed-manual searches for bugs with a dislodging spray is the standard method used for detecting infestations but it has limited sensitivity and precision [50], [51]. Its sensitivity for detecting domestic *T. infestans* infestations reached 70–77% in the dry Chaco [52], but likely declines at very low bug densities. At the expense of increased cost, repeated searches on the same site may be used to estimate a probability of bug detection that later can be incorporated in statistical modeling [53]. Information on the presence and abundance of animal hosts and domestic use of insecticides (both varying seasonally) based on householders' reports is subject to recall bias and therefore is rather imprecise. These issues probably underlie the limited fit of logistic and negative binomial models. Multiple regression analyses only show that the variability registered in the explanatory variables is not related strongly enough to the variability registered in the outcome variables (infestation or bug abundance). A statistically insignificant result may reflect that such explanatory and outcome variables are truly uncorrelated, or that there is not enough variability registered in them so as to detect any correlation. On the flip side, major strengths of this research effort are the detailed information at site level in a sizable number of house compounds from a well-defined rural area, analyzed with a multi-model inference approach that reduces overfitting.

Housing improvement is usually mentioned as the ultimate solution for domestic infestation [1], but in practice residual insecticide spraying remains virtually the only tool used for suppressing house infestation with triatomine bugs. Our results have implications in both directions: i) Housing improvement (including key peridomestic structures) should be promoted more widely and conducted more effectively to reduce refuge availability and bug infestations, rather than focusing solely or mainly on replacing some building materials (e.g., thatch by corrugated metal sheets). An urban-like house with brick-and-cement walls and corrugated-metal roof does not guarantee the absence of bugs, as shown in Figure S1B,H, since adequate refuges may still exist in walls, beds, furniture and stored goods. Conversely, mud houses may be kept free of *T. infestans* provided they are adequately maintained (Figure S1G), the presence of animal hosts in domiciles is minimized, and insecticides are used when needed [22], [26]. Inexpensive modifications of key peridomestic ecotopes, such as reducing refuge availability in ‘nideros’ or renovating them more often, may strongly reduce peridomestic populations of *T. infestans*; ii) Better housing with fewer refuges for bugs will likely reduce the chance of residual foci after insecticide spraying [8], [33] and facilitate community-based vector surveillance, and iii) Local and regional heterogeneities in the distribution of infestation and relevant factors (including human practices and socio-economic characteristics) need to be considered in order to prioritize and allocate insecticide spraying operations and vector surveillance more efficiently. The preference of *T. infestans* for certain hosts and types of refuge is strictly biological, but their occurrence in a house is also a social phenomenon that needs to be addressed more broadly. A more integral perspective that simultaneously considers social, economic and biological processes at local and regional scales, and the needs, possibilities and expectations of local populations is required for attaining effective and sustainable vector and disease control [47], [54]–[56].

## Supporting Information

Figure S1 **Model examples of degrees of refuge availability for** ***Triatoma infestans*** **in Pampa del Indio.** The scale of refuge availability ranges from 1 (no refuge at all) to 5 (abundant suitable refuges). A: Detail of a ‘nidero’ (elevated shelf for chickens to nest) against a mud wall, rated 5. Most refuges are provided by abundant deep cracks in the mud (with straw) wall below the shelf where the hen lays. Refuges are also provided by the piled bricks laid on mud beside the hen. B: Interior of a domicile with very deteriorated walls made of mud applied onto a wooden structure (part of the structure is noticeable in the picture) and a corrugated metal-sheet roof. Abundant refuges are found in wall cracks, particularly in the space between the wood poles and mud. The hanging clothes and bed also provide adequate refuge, although to a lesser degree than the deteriorated walls. Rated 5. C: Detail of a thatched ceiling with a thin layer of mud on its surface in a kitchen. Abundant refuges were provided mainly by the thatch and less so by cracks in the mud wall. Rated 5. D: An abandoned brick-made structure used as a ‘nidero’ on its top. The piled bricks with no mud or cement between them provide plenty of refuges for bugs to hide in although they are not very well protected. Rated 4. E: Detail of a wall with bricks laid on cement. The two holes indicated by the arrows provide fairly adequate refuges for bugs. This wall had several such holes and therefore was rated 4. Notice the fecal streaks of triatomine bugs (black spots) on the surface. F: Detail of a wall with bricks laid on mud with a hen nesting. Rated 4 because of several holes and cracks in the mud and between mud and bricks. G: Interior of a domicile with mud walls plastered with mud and a wooden ceiling (the roof made of corrugated metal sheets is not shown). Notice the excellent condition of some parts of the walls, especially those recently plastered (brown colored), and the few cracks in the older parts. Despite of the condition of such walls, household goods (especially the beds and the closet) provide some adequate refuge. Rated 3. H: Upper corner of the interior of a domicile showing a cement-plastered wall and a ceiling of wooden boards with tongue-and-groove joints. The wall provides no refuges for bugs but the small space left between the ceiling and wall allows bugs to access the air chamber between the ceiling and roof: rated 3. Notice the abundant bug feces (black spots and streaks) on wall surface. I: Detail of the wall of a goat corral made of piled logs and wire. Rated 2 because of the few cracks and bark in logs that provide some refuges though not very appropriate for *T. infestans* because they are exposed to the environment. J: A wired-fence goat corral with an elevated roof made with a few logs. Rated 1 because neither the logs nor the wire provided any adequate refuge for *T. infestans*. K: Detail of the wall of a kitchen, perfectly plastered and partially covered with tiles. The wall on its own would be rated 1; however, most kitchens usually contain other goods that can provide further refuge for bugs.
(PDF)

Table S1 **Reported domestic animal hosts resting or nesting according to ecotope.**
(PDF)

Table S2 **Number of sites per inhabited house according to ecotope and resident ethnic group.**
(PDF)

Text S1 **Explanation of script used for assessing the relative importance of different variables in relation to site-specific infestation and abundance of** ***T. infestans***
(PDF)

Script S1 **Script used for assessing the relative importance of different variables in relation to site-specific infestation and abundance of** ***T. infestans*** **.**
(TXT)

Alternative Language Text S1 **Text of article translated to Spanish by Juan M. Gurevitz, María Sol Gaspe, and Ricardo E. Gürtler.**
(PDF)
