# Supplementary material for: Factors Affecting Infestation by Triatoma infestans in a Rural Area of the Humid Chaco in Argentina: A Multi-Model Inference Approach
Source: PLoS Negl Trop Dis. 2011 Oct 18;5(10):e1349. doi: 10.1371/journal.pntd.0001349 (PMC3196485; doi:10.1371/journal.pntd.0001349)

Figure S1. Model examples of degrees of refuge availability for *Triatoma infestans* in Pampa del Indio.

The scale of refuge availability ranges from 1 (no refuge at all) to 5 (abundant suitable refuges). A: Detail of a 'nidero' (elevated shelf for chickens to nest) against a mud wall, rated 5. Most refuges are provided by abundant deep cracks in the mud (with straw) wall below the shelf where the hen lays. Refuges are also provided by the piled bricks laid on mud beside the hen.

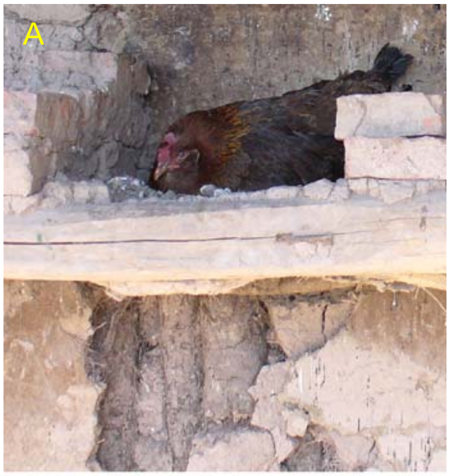

B: Interior of a domicile with very deteriorated walls made of mud applied onto a wooden structure (part of the structure is noticeable in the picture) and a corrugated metal-sheet roof. Abundant refuges are found in wall cracks, particularly in the space between the wood poles and mud. The hanging clothes and bed also provide adequate refuge, although to a lesser degree than the deteriorated walls. Rated 5.

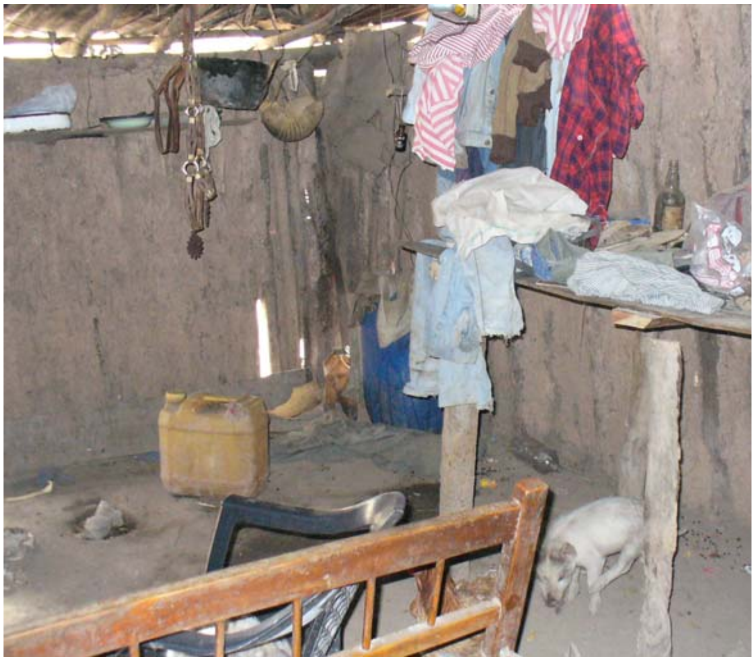

C: Detail of a thatched ceiling with a thin layer of mud on its surface in a kitchen. Abundant refuges were provided mainly by the thatch and less so by cracks in the mud wall. Rated 5.

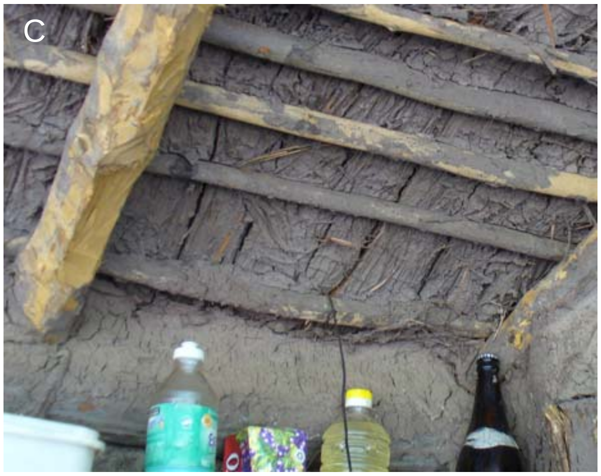

D: An abandoned brick-made structure used as a 'nidero' on its top. The piled bricks with no mud or cement between them provide plenty of refuges for bugs to hide in although they are not very well protected. Rated 4.

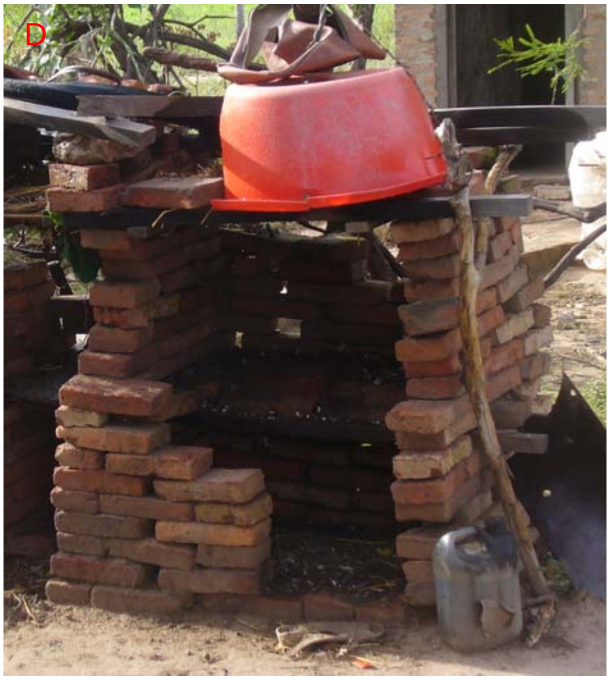

E: Detail of a wall with bricks laid on cement. The two holes indicated by the arrows provide fairly adequate refuges for bugs. This wall had several such holes and therefore was rated 4. Notice the fecal streaks of triatomine bugs (black spots) on the surface.

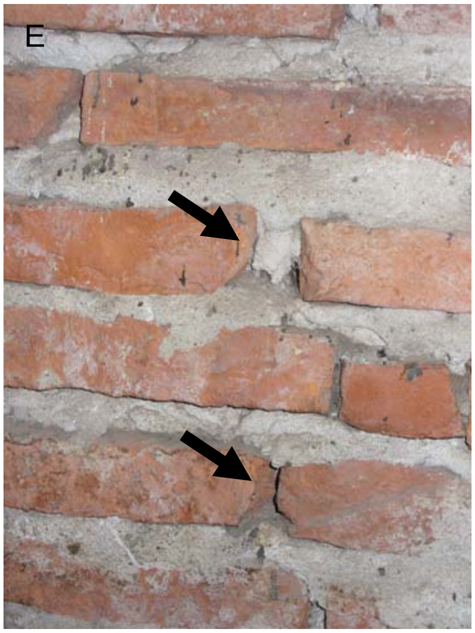

F: Detail of a wall with bricks laid on mud with a hen nesting. Rated 4 because of several holes and cracks in the mud and between mud and bricks.

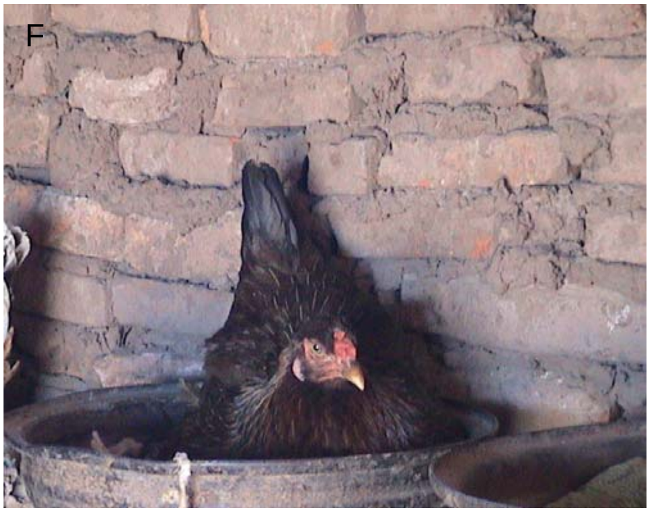

G: Interior of a domicile with mud walls plastered with mud and a wooden ceiling (the roof made of corrugated metal sheets is not shown). Notice the excellent condition of some parts of the walls, especially those recently plastered (brown colored), and the few cracks in the older parts. Despite of the condition of such walls, household goods (especially the beds and the closet) provide some adequate refuge. Rated 3.

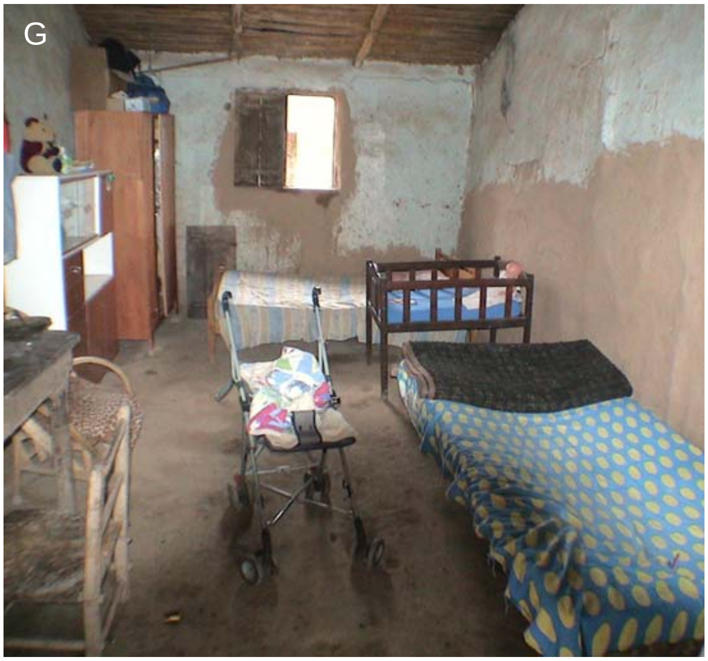

H: Upper corner of the interior of a domicile showing a cement-plastered wall and a ceiling of wooden boards with tongue-and-groove joints. The wall provides no refuges for bugs but the small space left between the ceiling and wall allows bugs to access the air chamber between the ceiling and roof: rated 3. Notice the abundant bug feces (black spots and streaks) on wall surface.

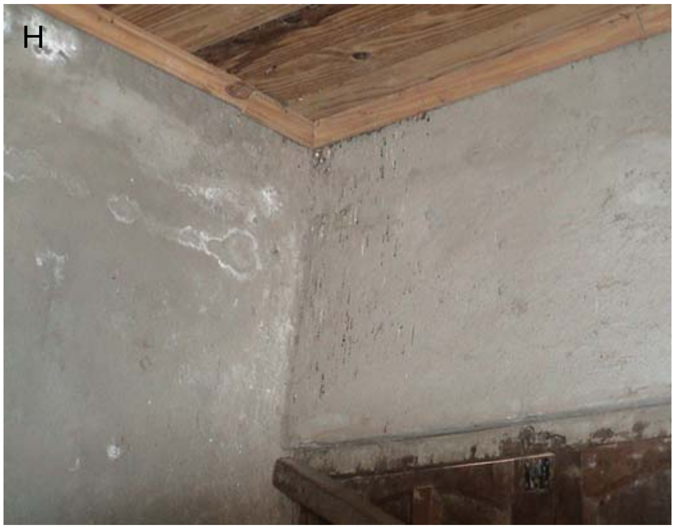

I: Detail of the wall of a goat corral made of piled logs and wire. Rated 2 because of the few cracks and bark in logs that provide some refuges though not very appropriate for *T. infestans* because they are exposed to the environment.

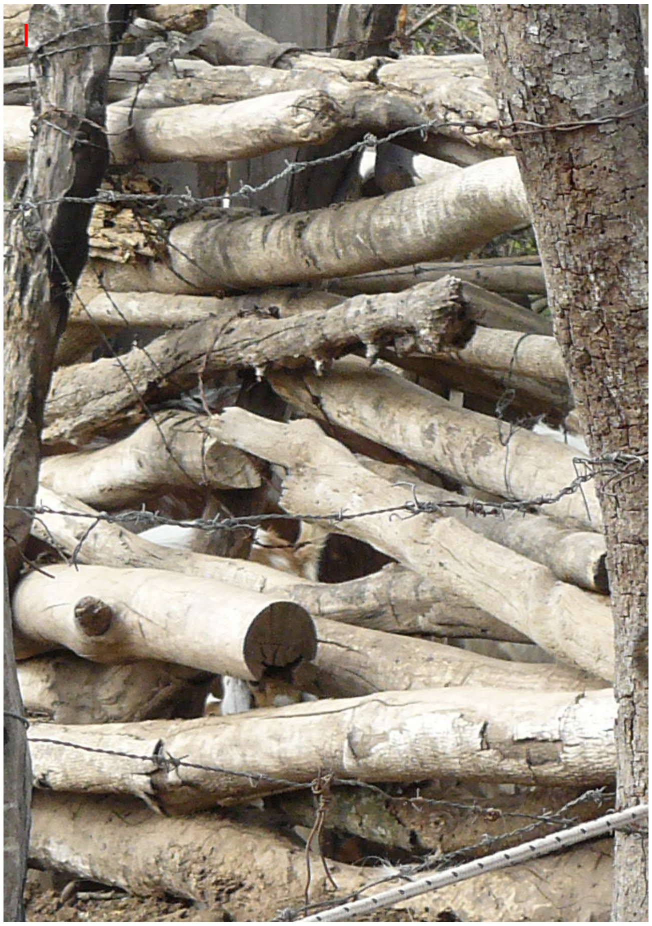

J: A wired-fence goat corral with an elevated roof made with a few logs. Rated 1 because neither the logs nor the wire provided any adequate refuge for *T. infestans*.

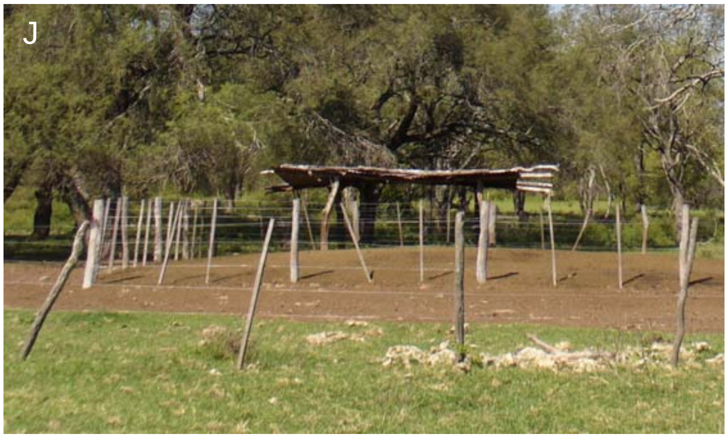

K: Detail of the wall of a kitchen, perfectly plastered and partially covered with tiles. The wall on its own would be rated 1; however, most kitchens usually contain other goods that can provide further refuge for bugs.

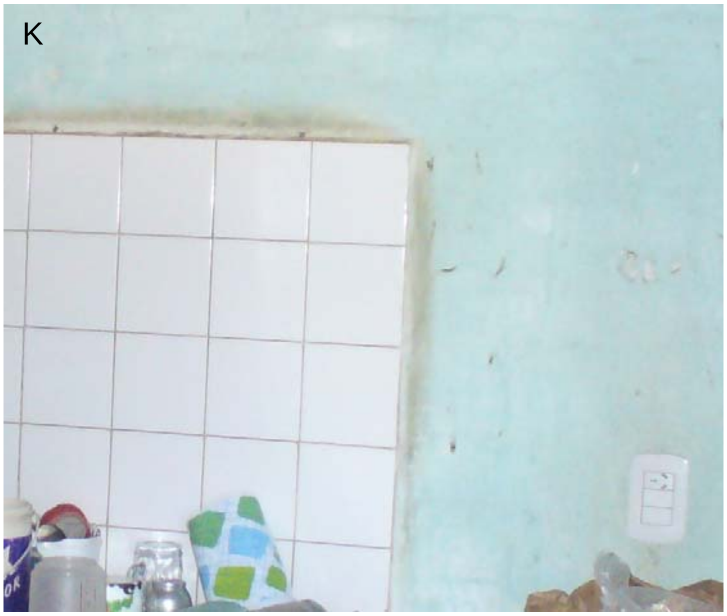

Supplement: Figure S1 — Model examples of degrees of refuge availability for Triatoma infestans in Pampa del Indio. The scale of refuge availability ranges from 1 (no refuge at all) to 5 (abundant suitable refuges). A: Detail of a ‘nidero’ (elevated shelf for chickens to nest) against a mud wall, rated 5. Most refuges are provided by abundant deep cracks in the mud (with straw) wall below the shelf where the hen lays. Refuges are also provided by the piled bricks laid on mud beside the hen. B: Interior of a domicile with very deteriorated walls made of mud applied onto a wooden structure (part of the structure is noticeable in the picture) and a corrugated metal-sheet roof. Abundant refuges are found in wall cracks, particularly in the space between the wood poles and mud. The hanging clothes and bed also provide adequate refuge, although to a lesser degree than the deteriorated walls. Rated 5. C: Detail of a thatched ceiling with a thin layer of mud on its surface in a kitchen. Abundant refuges were provided mainly by the thatch and less so by cracks in the mud wall. Rated 5. D: An abandoned brick-made structure used as a ‘nidero’ on its top. The piled bricks with no mud or cement between them provide plenty of refuges for bugs to hide in although they are not very well protected. Rated 4. E: Detail of a wall with bricks laid on cement. The two holes indicated by the arrows provide fairly adequate refuges for bugs. This wall had several such holes and therefore was rated 4. Notice the fecal streaks of triatomine bugs (black spots) on the surface. F: Detail of a wall with bricks laid on mud with a hen nesting. Rated 4 because of several holes and cracks in the mud and between mud and bricks. G: Interior of a domicile with mud walls plastered with mud and a wooden ceiling (the roof made of corrugated metal sheets is not shown). Notice the excellent condition of some parts of the walls, especially those recently plastered (brown colored), and the few cracks in the older parts. Despite of the [file pntd.0001349.s001.pdf]
