# Supplementary material for: Factors Affecting Infestation by Triatoma infestans in a Rural Area of the Humid Chaco in Argentina: A Multi-Model Inference Approach
Source: PLoS Negl Trop Dis. 2011 Oct 18;5(10):e1349. doi: 10.1371/journal.pntd.0001349 (PMC3196485; doi:10.1371/journal.pntd.0001349)
Supplement: Table S1 — Reported domestic animal hosts resting or nesting according to ecotope. (PDF) [file pntd.0001349.s002.pdf]

Table S1. Reported domestic animal hosts resting or nesting according to ecotope.

Ecotope	No. of sites	% of sites occupied by			
		Dogs or cats	Chickens sleeping	Chickens nesting	Fledglings
Domiciles	391	24.0	2.6	9.2	5.9
Kitchen or storerooms	379	23.5	4.2	12.1	13.7
Chicken coops	121	0	24.8	9.1	16.5
‘Nideros’	109	0	11.0	24.8	9.2
Other	41	4.9	0	0	2.4
Corrals	436	0	0.5	0.5	0.2
Latrines	239	0	0	0.4	0.4
Chicken trees	242	0	60.3	1.2	10.3
Ovens	161	0	0.6	4.3	0
Not used	46	2.2	6.5	4.3	4.3

People, goats, pigs, cattle and equines are excluded.
