# Supplementary material for: Factors Affecting Infestation by Triatoma infestans in a Rural Area of the Humid Chaco in Argentina: A Multi-Model Inference Approach
Source: PLoS Negl Trop Dis. 2011 Oct 18;5(10):e1349. doi: 10.1371/journal.pntd.0001349 (PMC3196485; doi:10.1371/journal.pntd.0001349)
Supplement: Table S2 — Number of sites per inhabited house according to ecotope and resident ethnic group. (PDF) [file pntd.0001349.s003.pdf]

Table S2. Number of sites per inhabited house according to ecotope and resident ethnic group.

Ecotope	Mean no./house ± SD		
	Creole	Toba	Total
Domiciles	1.2 ± 0.4	1.3 ± 0.5	1.2 ± 0.4
Kitchens and storerooms	1.4 ± 1.1	0.8 ± 0.7	1.7 ± 0.9
Corrals	1.6 ± 1.4	0.6 ± 0.7	2.0 ± 1.2
Chicken trees	1.0 ± 1.0	0.7 ± 0.7	1.4 ± 0.9
Latrines	0.7 ± 0.6	1.1 ± 0.6	1.1 ± 0.3
Ovens	0.6 ± 0.5	0.5 ± 0.5	1.1 ± 0.2
Chicken coops	0.5 ± 0.9	0.4 ± 0.7	1.4 ± 0.9
‘Nideros’	0.4 ± 0.6	0.2 ± 0.4	1.1 ± 0.3
Not used	0.2 ± 0.5	0.1 ± 0.3	1.2 ± 0.4
Other	0.2 ± 0.5	0.2 ± 0.5	1.2 ± 0.5
Total	7.9 ± 3.6	5.8 ± 2.4	7.4 ± 3.5
