# Supplementary material for: Factors Affecting Infestation by Triatoma infestans in a Rural Area of the Humid Chaco in Argentina: A Multi-Model Inference Approach
Source: PLoS Negl Trop Dis. 2011 Oct 18;5(10):e1349. doi: 10.1371/journal.pntd.0001349 (PMC3196485; doi:10.1371/journal.pntd.0001349)
Supplement: Text S1 — Explanation of script used for assessing the relative importance of different variables in relation to site-specific infestation and abundance of T. infestans (PDF) [file pntd.0001349.s004.pdf]

Explanation of script used for assessing the relative importance of different variables in relation to site-specific infestation and abundance of *T. infestans*

As an example, the script used for the negative binomial analysis for domiciles, kitchens and storerooms is presented. Other analyses were made similarly, replacing the variables considered, number of models and fitting commands accordingly.

Variables in data set:
ref_avail: refuge availability; integers from 1 to 5.
titmc: Triatoma infestans abundance according to timed-manual collections; integers ≥ 0.
tiq: Triatoma infestans presence according to timed-manual collections; categorical variable, valued 0 (absence) or 1 (presence).
Ethnic: ethnic group; categorical variable with two levels: “criollo” (reference level) or “toba”.
Insecticide: domestic insecticide use; categorical variable with two levels: “No” (reference level) or “Yes”.
Ecotope: ecotope of the site; categorical variable with two levels: “aDomi” (domiciles; reference level; the preceding ‘a’ makes that level to be considered the reference level because it appears first in alphabetical order) or “CoD” (kitchens and storerooms).
mud: mud present as a building material; categorical variable, valued 0 (absence) or 1 (presence).
wood: wood present as a building material; categorical variable, valued 0 (absence) or 1 (presence).
thatch: thatch present as a building material; categorical variable, valued 0 (absence) or 1 (presence).
cardboard: cardboard present as a building material; categorical variable, valued 0 (absence) or 1 (presence).
metal present as a building material; categorical variable, valued 0 (absence) or 1 (presence).
brick: brick present as a building material; categorical variable, valued 0 (absence) or 1 (presence).
dogcat: number of dogs and cats resting in the site.
fledg: number of fledglings resting in the site.
human: number of people living in the site.
ad_birds: number of adult birds resting or nesting in the site.

Description of script:

```
ref_availp=matrix(0,16383,2)
```
generates a matrix of 16383 rows and two columns for parameters (thus “p” in its name) of variable “ref_avail”. And so on for all other variables.

```
mod=glm.nb(titmc~ref_avail+Insecticide+human+ad_birds+dogcat+fledg+Ecotope+car
dboard+mud+thatch+brick+metal+wood+Ethnic,maxit=100)
```
runs the glm.nb command (negative binomial model fitting), assigning the output to “mod”. For details, refer to R help.

```
aic=AIC(mod)
```
keeps the AIC value of the fitted model in the vector “aic”.

```
ref_availp[1,]=coefficients(summary(mod))[names(mod$coefficients)=="ref_avail"
,1:2]
```

keeps the parameters (regression coefficient and variance) of variable "ref_avail" in the model number 1.

```
InsecticideYesp[1,]=coefficients(summary(mod))[names(mod$coefficients)=="Insec
ticideYes",1:2]
```

stores the parameters (regression coefficient and variance) of the level "Yes" of variable "Insecticide" in the model number 1.
And so on for all other variables.

After this first model is run, the next model is run and its parameters stored, and so on until all models are run.

```
ref_availp[is.na(ref_availp)]=0
```

replaces all empty cells ("na") with 0 in the matrix of the parameters of variable "ref_avail". And so on for all other matrices of variables.

```
weight=exp(-0.5*(aic-min(aic)))
weight=weight/sum(weight)
```

calculates the AIC weight of each model run according to the formula:

$$w_i = \frac{e^{\left(-\frac{1}{2}(AIC_i - AIC_{max}\right)}}{\sum_{r=1}^{R} e^{\left(-\frac{1}{2}(AIC_r - AIC_{max}\right)}}$$

where w_i is the weight of model i, AIC_i is the AIC value of model i, AIC_{max} is the maximum value of AIC among all the models considered, and R is the total number of models considered.

```
wi_vars<-weight%*%as.matrix(matriz)
```

generates the matrix "wi_vars" by multiplying the "weight" vector by the matrix "matriz" indicating the presence or absence of each variable in each of the models considered.

```
param_modelo=matrix(0,14,2)
```

generates the two-column matrix for the 14 variables considered in order to store the average parameters of each variable.

```
param_modelo[1,1]<-ref_availp[,1]%*%weight
```

calculates the AIC-weighted average of the parameters of variable "ref_avail". And so on for all other variables.
