# Supplementary material for: Factors Affecting Infestation by Triatoma infestans in a Rural Area of the Humid Chaco in Argentina: A Multi-Model Inference Approach
Source: PLoS Negl Trop Dis. 2011 Oct 18;5(10):e1349. doi: 10.1371/journal.pntd.0001349 (PMC3196485; doi:10.1371/journal.pntd.0001349)
Supplement: Alternative Language Text S1 — Text of article translated to Spanish by Juan M. Gurevitz, María Sol Gaspe, and Ricardo E. Gürtler. (PDF) [file pntd.0001349.s006.pdf]

Factores que afectan la infestación por *Triatoma infestans* en un área rural del Chaco húmedo en Argentina: un abordaje de inferencia multi-modelo

Juan M. Gurevitz,[1] Leonardo A. Ceballos,[1] María Sol Gaspe,[1] Julián A. Alvarado-Otegui,[1] Gustavo F. Enríquez,[1] Uriel Kitron,[2] Ricardo E. Gürtler[1,*]

1 Laboratorio de Eco-Epidemiología, Departamento de Ecología, Genética y Evolución, Universidad de Buenos Aires, Buenos Aires, Argentina. **2** Dept. of Environmental Studies, Emory University, Atlanta, GA 30322, USA.

* E-mail: gurtler@ege.fcen.uba.ar

Autor para correspondencia: Ricardo E. Gürtler, Laboratorio de Eco-Epidemiología, Departamento de Ecología, Genética y Evolución, Universidad de Buenos Aires, Ciudad Universitaria, 1428 Buenos Aires, Argentina. Tel/Fax: +54-11-4576-3318.

Resumen

Contexto

La transmisión de *Trypanosoma cruzi* por *Triatoma infestans* persiste como un importante problema de salud pública en la ecorregión del Gran Chaco, donde la comprensión de los determinantes de la infestación de las casas es todavía limitada. Se realizó un estudio transversal para modelar los factores que afectan la presencia y la abundancia de *T. infestans* en los sitios de las casas (‘house compounds’) en un área rural definida en el Chaco húmedo argentino.

Metodología/Resultados principales

Se halló *Triatoma infestans* en 45,9% de las 327 casas habitadas pero sólo en 7,4% de los 2.584 sitios evaluados en estas casas, a pesar de que el último rociado de insecticidas había ocurrido 12 años antes. Los sitios infestados estaban agregados significativamente a distancias de 0,8-2,5 km. Los ecotopos más frecuentemente infestados fueron los domicilios, cocinas, depósitos, gallineros y nidos; los corrales raramente estuvieron infestados. Los domicilios con paredes de barro y techo de paja o chapa de cartón alquitranado estaban más frecuentemente infestados (32,2%) que los domicilios con paredes de ladrillo y cemento y techos metálicos (15,1%). Se utilizó un abordaje de inferencia multi-modelo utilizando el criterio de información de Akaike para evaluar la importancia relativa de cada variable considerada corriendo todos los modelos (17.406) generados de todas las combinaciones posibles de las variables. La disponibilidad de refugios, la construcción con cartón y la cantidad de hospedadores (personas, perros, gatos y aves) por sitio resultaron positivamente asociados con la infestación y la abundancia de triatominos, mientras que el uso de insecticidas por los moradores mostró una asociación negativa. La etnia (criolla o toba), ajustada por los demás factores, mostró una leve o nula asociación con la infestación y la abundancia.

Conclusiones/Implicancias

Para eliminar la infestación en forma sostenible se requiere la promoción y efectiva implementación del mejoramiento de la vivienda (incluyendo sitios peridomésticos clave) junto con un uso adecuado de insecticidas y mejor manejo de los hospedadores. Menos refugios se traducirían en menos focos residuales tras el rociado con insecticidas y facilitaría la vigilancia vectorial. Para alcanzar un control vectorial y de la enfermedad efectivo y sostenible se necesita una perspectiva más integral, que considere simultáneamente los procesos sociales, económicos y biológicos a nivel local y regional, junto con las posibilidades, necesidades y expectativas de la población local

Resumen del autor

La transmisión vectorial de la enfermedad de Chagas continúa siendo un importante problema de salud pública en parte de América Latina. *Triatoma infestans* es el principal vector en los países del Cono Sur, particularmente en la ecorregión del Gran Chaco donde el control con insecticidas residuales ha logrado sólo un moderado e irregular impacto. A fin de contribuir a mejores estrategias de control, analizamos los factores asociados con la presencia y abundancia de *T. infestans* en 327 casas habitadas en un área rural definida sin historia reciente de control vectorial en el Chaco húmedo argentino. Se encontraron triatominos principalmente en los domicilios, cocinas, depósitos, gallineros y nidos, particularmente donde había refugios adecuados y hospedadores (personas, perros, gatos o aves). Los domicilios hechos de barro fueron los más frecuentemente infestados, aunque domicilios de ladrillo y cemente, incluso en buenas condiciones, también estuvieron infestados. La disponibilidad de refugios y hospedadores para *T. infestans* son objetivos clave para el control vectorial. Diferencias entre parajes vecinos de hasta diez veces en el nivel de infestación y diferencias en los factores relevantes para la presencia de *T. infestans* respecto a otras área del Gran Chaco sugieren que el manejo de hospedadores, las técnicas de construcción y el uso de insecticidas tienen que ajustarse a las características ambientales, socio-económicas y climáticas locales.

Introducción

La transmisión de *Trypanosoma cruzi*, el agente etiológico de la enfermedad de Chagas, por insectos hematófagos de la familia Triatominae continúa siendo un problema de salud pública en muchas comunidades rurales y algunas periurbanas de América Latina [1]. Durante décadas, las acciones de control vectorial consistieron mayormente en la aplicación de insecticidas residuales; su implementación ha sido geográficamente heterogénea y sus impactos sobre la transmisión de *T. cruzi*, variables. Además de las características regionales particulares y las dificultades inherentes al control vectorial, la transmisión de *T. cruzi* ocurre principalmente en poblaciones humanas marginadas [2].

Triatoma infestans no es la excepción. Esta especie infesta principalmente viviendas rurales pobres y las estructuras asociadas a ellas. Si bien el rango de distribución de *T. infestans* ha sido ampliamente reducido y la transmisión del parásito interrumpida en Brasil, Chile y Uruguay [3], la aplicación de insecticidas piretroides ha tenido un impacto moderado e irregular en el núcleo de su distribución donde se han encontrado serios obstáculos para la eliminación de este vector [1,4,5]. Esta área corresponde principalmente a la ecorregión del Gran Chaco, una llanura semiárida de 1,1 millones de km^2 que abarca grandes porciones del norte y centro de Argentina, sudeste de Bolivia y centro y oeste de Paraguay [6]. La población humana consiste principalmente en criollos y diferentes grupos indígenas distribuidos en forma dispersa en las zonas rurales, con escaso acceso a la muy pobre infraestructura existente, incluyendo servicios de salud débiles e instituciones con políticas provinciales y federales inconsistentes [7]. Estos factores modifican la efectividad de las operaciones de control vectorial en un área donde *T. infestans* se desarrolla rápidamente y alcanza altas abundancias y las condiciones ambientales disminuyen la efectividad de los insecticidas piretroides en algunas de las estructuras peridomésticas más fuertemente infestadas [8,9]. Las estructuras peridomésticas usualmente desempeñan un rol fundamental en el mantenimiento de poblaciones abundantes de triatominos de distintas especies en la cercanía de los domicilios [8,10-16].

La comprensión de los factores asociados a la infestación ayudaría a dilucidar cómo se materializan las dimensiones sociales del problema; identificar posibles objetivos de intervención, y predecir los riesgos de transmisión del parásito. De manera general, *T. infestans* depende de la presencia de tres características biofísicas básicas para establecer colonias exitosas: i) hospedadores de sangre caliente, ii) hábitats y condiciones climáticas adecuadas, y iii) ausencia de exposición a insecticidas efectivos. Los humanos, perros, gatos y gallinas han sido identificados reiteradamente como importantes hospedadores domésticos [17]. Los hábitats adecuados para los triatominos se han asociado frecuentemente con características estructurales de las viviendas y las estructuras peridomésticas: condiciones precarias de construcción, grietas en las paredes, viviendas de barro y paja y determinadas estructuras peridomésticas, tales como corrales de cabras o cerdos y gallineros [10,12,18-20]. La

aplicación de insecticidas de baja concentración por parte de los pobladores también se asoció negativamente con la infestación [21,22]. Rara vez se ha abordado simultáneamente la relación entre estas variables biofísicas y la infestación en un gran número de viviendas utilizando métodos estandarizados de captura de insectos, información detallada en unidades de hábitat biológicamente significativas, y métodos analíticos robustos que reduzcan el sesgo en la selección de variables. Un abordaje de inferencia multi-modelo resulta útil para este objetivo [23] y no ha sido aplicado a los vectores de la enfermedad de Chagas.

A pesar de la amplia distribución de *T. infestans*, las variables asociadas con la infestación de las casas se abordaron meticulosamente en pocas zonas: en el Cerrado brasileño [10,24] y en la región seca (oeste) del Gran Chaco [8,21,22]. Sin embargo, la ecología de *T. infestans* y los patrones de transmisión del parásito podrían variar entre ecorregiones dependiendo del clima, el ambiente, las prácticas humanas y las características étnicas. Raramente se han aportado descripciones detalladas de los elementos clave que favorecen la infestación de determinados hábitats. Como parte de un proyecto de investigación multi-sitio sobre la eco-epidemiología y control de *T. infestans* en el Gran Chaco, el presente estudio tiene como objetivo modelar las variables asociadas con la presencia y la abundancia de *T. infestans* en un área rural bien definida del Chaco argentino 12 años después de la última campaña de rociado con insecticidas piretroides.

Materiales y Métodos

Área de estudio y Población

Este estudio fue llevado a cabo en una porción (450 km^2) del municipio de Pampa del Indio (25º 55'S 56º 58'O), provincia de Chaco, Argentina (Figura 1A, recuadro), ubicada en el Chaco húmedo (este), cercano a la transición con el Chaco seco (oeste). Esta área fue seleccionada para llevar a cabo el proyecto en base a datos recolectados por el Programa de Chagas de Chaco, que indicaban altos niveles de infestación. En base a un estudio piloto en toda el área rural de Pampa del Indio, en el cual se inspeccionó la infestación por triatominos en una muestra de viviendas (11%) seleccionadas de manera sistemática, seleccionamos una zona bien definida que presentaba niveles de infestacion levemente mayores que el resto y con más de 300 casas contiguas separadas ≥ 1 km de los demás parajes vecinos.

El área de estudio incluyó 353 casas y varios edificios públicos de 13 comunidades rurales vecinas (Figura 1). El clima es continental, cálido, con lluvias principalmente en verano. La temperatura media anual es 22,8 ºC (media mínima y máxima, 16,9 y 29,3 ºC, respectivamente). Las precipitaciones anuales históricas han sido de 954 mm, mientras que en 2008 y 2009 una severa sequía afectó la región. El paisaje es llano y comprende un mosaico de parches cultivados, bosque seco nativo con variado nivel de degradación, y ocasionalmente cuerpos de agua y pantanos.

Los dos grupos étnicos principales son criollos y tobas. Los tobas representan el 24% de los 1.187 habitantes del área de estudio y ocupan el 16% de las viviendas. Los criollos son descendientes de europeos y en general tuvieron un alto grado de mezcla con indígenas en generaciones pasadas. La mayoría de los criollos migraron al área durante los últimos 50-100 años desde provincias cercanas o desde Europa. Los tobas –el único grupo indígena en el área- eran tradicionalmente nómades o cazadores-recolectores semi-nómades. Siguiendo la colonización local en la década de 1920s comenzaron a depender cada vez más de la agricultura, trabajos informales temporarios y, desde hace unas pocas décadas, programas de asistencia social estatales [25]. Las autoridades locales reportaron contar con aproximadamente 5.000 beneficiarios de programas de asistencia social entre los ~14.000 habitantes de todo el municipio, incluyendo tobas y criollos. Los residentes rurales desarrollan mayormente una economía de subsistencia y suelen sembrar algodón, maíz, calabaza, melón o criar ganado (principalmente cabras, pero también vacas y ocasionalmente ovejas). El hospital más cercano se encuentra en el pueblo de Pampa del Indio (~5.000 habitantes), a 10-45 km del área de estudio por rutas de tierra. En 1996 se llevó a cabo la última campaña masiva de rociado con insecticidas por personal de control de vectores, a excepción de una pocas viviendas que fueron tratadas en 2006 por los pobladores o el personal del hospital; no se realizó ninguna campaña educativa específica o sistemática en relación a la enfermedad de Chagas en esta área.

El área de estudio abarcó 327 casas ('house compounds') habitadas, incluyendo todos sus sitios domésticos y peridomésticos. Se regirtraron 26 casas deshabitadas o abandonadas y 37 edificios públicos (incluyendo 11 escuelas, cinco centros de atención primaria de la salud, y varios templos y centros comunitarios). El concepto de casa incluye el domicilio y todos los sitios dentro del área peridoméstica (es decir, el peridomicilio) –en general una letrina, un depósito, una cocina, un horno, uno o más corrales, uno o más sitios para las gallinas y otras aves de corral (árboles, gallineros, nidos) (Figura 2A,B). Un sitio fue definido como cualquier estructura individual construida o utilizada por los habitantes, que puede proveer refugio a triatominos. El ecotopo (variable categórica) fue definido como un sitio caracterizado por determinadas estructuras y usos típicos (por ej., domicilio, depósito, gallinero, etc.). Los nidos presentaron frecuentemente una estructura distintiva llamada 'nidero' (Figura 2C). Ésta consiste en una plataforma elevada, hecha de madera o a veces de ladrillos donde las gallinas y, ocasionalmente, pavos o patos anidan. La mayoría de los domicilios eran viviendas de barro y paja con techos de chapa de metal o cartón alquitranado.

Encuesta de hogares

Del 4-15 de septiembre y 24 de octubre-17 de noviembre de 2007 se realizó un estudio transversal de todas las viviendas del área de estudio junto con personal local (agentes de salud, personal de control de vectores) para explicar a los habitantes los objetivos de la investigación en un lenguaje claro y simple, y para solicitarles permiso para acceder a sus casas, siguiendo el protocolo aprobado por IRB No. 00001678 (registrado en NIH). Para cada casa se registró el nombre completo del jefe de familia y las cantidades de habitantes de 0-4 años y de 5-14 años. Se realizó un mapa con la ubicación espacial de las diferentes estructuras de cada casa. A cada estructura se le asignó un código único individual de acuerdo a su uso para poder identificarla en las sucesivas visitas y asociarla con los triatominos recolectados. Se registró la localización geográfica de cada sitio utilizando un receptor GPS (Trimble GeoXM o Garmin Legend). Con el consentimiento de los moradores, se colocó una placa de aluminio numerada en cada una de las casas y edificios públicos. En cada hogar se entregó una bolsa plástica rotulada con cierre a presión para que conservaran cualquier triatomino que pudieran capturar en los sitios domésticos o peridomésticos luego del rociado con insecticidas. Se le pidió a los moradores que conservaran los insectos hasta nuestra siguiente visita y se los instruyó en cómo recolectarlos sin incurrir en un riesgo de contaminación.

Para cada sitio se registraron los materiales utilizados en la construcción del techo y las paredes, la presencia de paredes revocadas, el estado de la superficie de las paredes, el material del revoque, el ecotopo y sus características físicas. La disponibilidad de refugios apropiados para *T. infestans* se determinó visualmente por un miembro del grupo de investigación experimentado en la búsqueda de triatominos; se clasificó de 1 (ausencia de refugios) hasta 5 (abundante cantidad de refugios). Se encuestó al jefe de familia (o algún otro adulto de la casa) acerca de la cantidad y tipo de animales doméstico presentes, sus lugares de descanso y el uso doméstico de insecticidas (tipo, frecuencia, modo y fecha de la última aplicación). La información sobre el uso de insecticidas, el ecotopo, los materiales de construcción, la disponibilidad de refugios, el número de personas y hospedadores y sus lugares de descanso fue registrada en abril, agosto y diciembre de 2008. Debido a que la cantidad de hospedadores animales varía estacionalmente, los datos recabados en diciembre de 2008 fueron utilizados para describir la cantidad de hospedadores en el relevamiento inicial llevado a cabo en octubre de 2007. Esto podría introducir algunas imprecisiones, en particular en sitios raramente utilizados por uno o muy pocos hospedadores, tal como algunas gallinas u otras aves de corral que anidan en un domicilio o depósito, ya que esto podría variar de un año a otro. En el caso de las demás variables sin variación estacional, se utilizaron los datos recabados en abril de 2008; en el caso que existieran valores faltantes, se utilizaron los datos obtenidos en el siguiente relevamiento.

Para evaluar si la determinación visual de la disponibilidad de refugio de un sitio variaba entre diferentes observadores, y luego de realizar una estandarización preliminar de los criterios en dos casas, seis evaluadores experimentados familiarizados con el área de estudio registraron independientemente la disponibilidad de refugio de 145 sitios pertenecientes a 45 casas. Estos evaluadores fueron agrupados en tres equipos que clasificaron los sitios inspeccionados durante un día de relevamiento.

Relevamiento entomológico

Simultáneamente con la encuesta de hogares, todos los sitios de cada una de las casas fueron inspeccionados para determinar la presencia de triatominos por el método de hora-hombre por dos evaluadores calificados de los programas nacional o provincial de control de vectores utilizando tetrametrina al 0,2% (Espacial, Argentina) como agente desalojante. Los domicilios fueron inspeccionados por una persona durante 20 minutos mientras que cada sitio peridoméstico fue revisado por una persona durante 15 minutos. En la práctica, la mayor parte de las búsquedas duraron menos tiempo debido a que el sitio se inspeccionaba completamente antes del tiempo estipulado. Por lo tanto, la duración de las búsquedas en los domicilios y en los demás ecotopos fue similar y el esfuerzo de captura se redondeó a 0,25 hora-hombre por sitio. Los edificios públicos fueron inspeccionados de la misma manera. En varias casas también se recolectaron triatominos luego del tiempo estipulado de búsqueda (capturas post hora-hombre), durante las aplicaciones de insecticidas o por los moradores unos días después de las búsquedas manuales o del rociado con insecticidas. Estas fuentes adicionales de insectos sólo se consideraron como medidas cualitativas de infestación. Los triatominos recolectados por los moradores se consideraron sólo cuando constaba la fecha y el lugar de captura. Inmediatamente después del relevamiento entomológico, todos los sitios de cada casa fueron rociados con dosis estándar (25 mg/m^2) de deltametrina floable (K-Othrin, Bayer) por personal de control de vectores. Los triatominos recolectados fueron transportados al laboratorio de campo en bolsas plásticas rotuladas con el sitio de captura, identificados taxonómicamente y contados por especie, estadio o sexo [26].

Análisis de datos

Todos los resultados se informan sólo para casas habitadas, excepto que se indique lo contrario; las casas deshabitadas y los edificios públicos no se hallaron infestados. La prevalencia de infestación y de colonización por *T. infestans* se calculó tanto a nivel de sitio como de casa. Un sitio se consideró infestado cuando se capturó en él al menos un adulto o una ninfa vivos de *T. infestans* por el método de hora-hombre. Si se capturó al menos una ninfa viva, el sitio también se consideró colonizado. La abundancia de triatominos se calculó como el número de ejemplares vivos de *T. infestans* capturados por medio del método de hora-hombre en un determinado sitio. La prevalencia de infestación (o colonización) a nivel de sitio se calculó como el número total de sitios infestados (o colonizados) dividido por el número total de sitios inspeccionados. La prevalencia de infestación (o colonización) a nivel de casa se calculó como el número total de casas infestadas (o colonizadas) dividido por el número total de casas inspeccionadas.

La relación entre los factores estudiados y la infestación o la abundancia de triatominos a nivel de sitio se evaluó utilizando modelos de regresión logística y binomial negativa, respectivamente. Se eligió la regresión binomial negativa en lugar de la de Poisson porque la abundancia de triatominos tenía una distribución agregada. Esto se reflejó en valores extremadamente más altos del criterio de información de Akaike (AIC) para los modelos de Poisson que para los binomiales negativos (datos no mostrados). El análisis se restringió a los ecotopos más frecuentemente infestados (es decir, domicilios, depósitos o cocinas, gallineros y nideros) porque en algunos casos las iteraciones del procedimiento de máxima verosimilitud no convergían cuando se incluían ecotopos raramente infestados en los modelos binomiales negativos. Los análisis de regresión se realizaron a nivel de sitio (es decir, cada observación individual en las regresiones representaba un sitio).

Se realizaron dos grupos diferentes de análisis según las características típicas de los ecotopos a fin de tomar en cuenta el efecto que tenía la estructura física de los sitios sobre la detectabilidad de triatominos y, por lo tanto, la comparabilidad de los resultados. Un grupo de análisis abarcó los ecotopos típicamente con paredes propiamente dichas y techo (es decir, domicilios, cocinas y depósitos); el segundo grupo abarcó los pequeños sitios (peridomésticos) utilizados por las gallinas y otras aves de corral (es decir, gallineros y nideros). En ambos casos la variable de respuesta fue la infestación a nivel de sitio para las regresiones logísticas y la abundancia de triatominos para las regresiones binomiales negativas. Para los domicilios, cocinas y depósitos las variables predictivas incluidas en los modelos fueron la etnia, el uso de insecticidas por los moradores, el ecotopo (con dos categorías, tomando a los domicilios como la de referencia), los materiales de construcción (cada material fue considerado como una variable diferente

ya que varios de ellos podían estar presentes en un mismo sitio), la disponibilidad de refugios, las cantidades de personas, perros o gatos, aves (mayormente gallinas) y pollitos. Para los gallineros y nideros se consideraron las mismas variables predictivas excepto por la etnia (debido a pocos sitios de estos ecotopos en casas tobas), el uso de insecticidas (básicamente restringido a los domicilios, cocinas y depósitos), y las cantidades de personas y perros o gatos (ya que no fueron informados en estos ecotopos).

Los coeficientes de correlación fueron bajos ($-0.2 < r < 0.2$) para todos los pares de variables, excepto: barro y disponibilidad de refugios ($r = 0.38$), barro y paja ($r = 0.39$), metal y ladrillo ($r = 0.32$), domicilios y madera ($r = -0.27$); todos estos coeficientes fueron significativos estadísticamente ($P < 0.05$). Sin embargo, dada la posibilidad de dependencias lineales involucrando más de dos variables (es decir, multicolinealidad) y su posible efecto perjudicial sobre la estimación de parámetros, se calcularon los números de condición para las matrices de variables predictivas [27]. El mayor número de condición fue 8, indicando una dependencia débil entre las variables que no debería implicar problema alguno de estimación [27].

Se eligió un abordaje de inferencia multi-modelo ('multi-model inference approach') basado en el AIC para evaluar la importancia relativa (IR) de cada variable de acuerdo a Burnham y Anderson [23]. Este abordaje permite ordenar cuantitativamente la contribución relativa de cada variable al ajuste del modelo dados las variables y los modelos considerados, a la vez que contribuye a reducir el sobreajuste ('overfitting'). Se corrieron todos los modelos considerando cada una de las combinaciones posibles de las variables predictivas: 14 variables generaron 16.383 modelos para los domicilios, cocinas y depósitos, y 10 variables generaron 1.023 modelos para los gallineros y nideros. Consideramos todas las variables como posibles candidatas ya que no contábamos con suficiente evidencia sobre su importancia relativa al considerarlas simultáneamente. Por lo tanto, los modelos considerados no fueron modelos alternativos que surgieron durante la experiencia. Se calculó la verosimilitud relativa (es decir, los pesos de Akaike) de cada modelo como el cociente entre el logaritmo de la verosimilitud ('log-likelihood') del modelo en cuestión dividido por la suma total de los logaritmos de la verosimilitud de todos los modelos considerados. La IR de cada modelo se calculó como la suma de los pesos de Akaike de todos los modelos en los que la variable en cuestión estaba presente. El máximo valor que puede tener la IR es 1, representando la máxima importancia relativa al grupo de variables consideradas; IR = 0 representa una importancia nula respecto a las variables consideradas. Las estimaciones de los parámetros de cada variable se obtuvieron del promedio del valor del parámetro en cuestión en cada modelo donde la variable estaba presente, ponderado por el peso de Akaike del respectivo modelo. Se utilizó la prueba de bondad de ajuste de Hosmer-Lemeshow para evaluar la calidad global del ajuste de los modelos logísticos, utilizando los coeficientes promediados y agrupando los datos en 10 grupos de igual tamaño; el área bajo la curva característica operativa del receptor (curva ROC); la sensibilidad y la especificidad utilizando como valor de corte la prevalencia observada de infestación para cada conjunto de datos (17,7% para domicilios, cocinas y depósitos; 14,8% para gallineros y nideros). Los análisis y cálculos se realizaron en R 2.7.0 [28]; los programas ('scripts') con los comandos para ejecutar los modelos y los correspondientes cálculos se prepararon utilizando un macro de Visual Basic en Microsoft Excel (Texto S1 y Script S1); el ajuste de los modelos se evaluó en Stata 9.0 [29].

La distribución espacial de los sitios infestados se evaluó utilizando el método de marcas al azar ('random labeling') con la función de correlación pareada ('pair-correlation function') implementadas en Programita [30]. La función de correlación pareada $g_{12}(r)$ evalúa si la cantidad de puntos del patrón 2 dentro de un anillo de radio r y un dado ancho, cuyo centro se sitúa sobre cada punto del patrón 1, corresponde en promedio a un patrón al azar (es decir, a un proceso homogéneo de Poisson; $g_{12}(r) = 1$), agregación de 2 alrededor de 1 ($g_{12}(r) > 1$), o regularidad de 2 respecto a 1 ($g_{12}(r) < 1$). El método de marcas al azar evalúa la distribución espacial de los puntos pertenecientes a un dado patrón –relativo a los demás puntos pertenecientes a ese u otro patrón– tomando en cuenta la distribución espacial de todos los puntos. Varias características propias de cada sitio afectan la probabilidad que ellos tienen de estar infestados, más allá de la cercanía o no a otros sitios infestados. Por lo tanto, en el presente caso el modelo nulo para reasignar la pertenencia de cada punto a cada patrón (y así generar las distribuciones nulas esperadas) tomó en cuenta la probabilidad de estar infestado de cada sitio según el modelo logístico con

todas las variables consideradas. El análisis espacial sólo incluyó sitios pertenecientes a los cuatro ecotopos más frecuentemente infestados (es decir, domicilios, depósitos y cocinas, gallineros y nideros). El tamaño de la cuadrícula para el análisis fue de 100 m; el ancho del anillo, 400 m; el radio máximo, 5 km; se corrieron 999 simulaciones y la 25ª simulación superior y la 25ª inferior definieron el rango de confianza ('confidence envelope') del 95%. Se utilizó una prueba de bondad de ajuste [30] para evaluar el ajuste global del patrón observado a la distribución esperada bajo el modelo nulo.

Para evaluar la concordancia entre los diferentes observadores en la estimación visual de la disponibilidad de refugios, se calculó el índice kappa de concordancia entre observaciones utilizando Stata 9.0 [29]. Esta medida de concordancia alcanza un valor de 1 cuando la concordancia entre las observaciones es completa y 0 cuando la concordancia registrada es similar a la esperada por azar. Valores > 0,60 pueden considerarse como una concordancia substancial a perfecta; valores < 0,40 representan una pobre concordancia más allá del azar, mientras que valores entre 0,40-0,60 implican una concordancia moderada [32].

Resultados

En total, se localizaron, ubicaron sobre un mapa e inspeccionaron en cuanto a su infestación 2.584 sitios domésticos o peridomésticos. La búsqueda manual de *T. infestans* por el método de hora-hombre encontró infestado el 39,8% de las 327 casas habitadas y sólo el 7,4% de todos los sitios evaluados; en 30,8% de las casas habitadas había colonias. El 25,9% de los domicilios y el 26,2% de los peridomicilios estaban infestados por *T. infestans*. Se halló *Triatoma sordida* en 18,3% de las casas, en 1,2% de los domicilios y en 17, 6% de los peridomicilios. En total, se capturaron 2.062 individuos de *T. infestans* y 331 de *T. sordida* por el método de hora-hombre. Considerando conjuntamente los resultados de todos los métodos de captura, la infestación por *T. infestans* alcanzó el 45,9% de las casas.

Ecotopos: infestación y características de construcción

Los ecotopos más frecuentemente infestados fueron los domicilios (25,8%), los depósitos o cocinas (15,4%), los gallineros (13,2%) y los nideros (18,2%), mientras que < 1,0% de los corrales (fuesen para cabras, cerdos o vacas), los baños, los árboles usados por las aves domésticas y otros ecotopos estaban infestados (Figura 3). La abundancia relativa de *T. infestans* fue máxima en los nideros, seguidos por los depósitos y cocinas, y resultó muy variable dentro y entre ecotopos (Figura 4). Entre los ecotopos frecuentemente infestados, la infestación en los gallineros fue la más variable. Los corrales de cabras y otros tipos de corral raramente estuvieron infestados. En ellos la abundancia de triatominos también fue muy variable pero esto se debió a la gran cantidad (80) de insectos capturados en uno solo de los cuatro sitios infestados.

La mayoría de los domicilios estaban construidos parcial o totalmente con barro en sus paredes (55,2%) y chapa metálica en sus techos (66,2%) (Tabla 1). Entre los domicilios con techo metálico, una importante porción tenía parte de su techo también con paja (14%) o chapa de cartón alquitranado (7%) y cielorrasos de madera (14%) o cañas (2%), proveyendo todos estos materiales adecuado refugio para triatominos. El barro, mezclado con pasto, se utilizaba principalmente aplicado sobre una estructura de madera, constituyendo la pared (Figura S1A,G) y, menos frecuentemente, se usaba como cemento para levantar paredes de ladrillo cocido (Figura S1F). Las paredes deterioradas de barro solían tener grandes grietas al separarse el barro de los palos de madera del armazón (Figura S1B); el barro utilizado como revoque también generaba grietas con el tiempo, aunque mucho más pequeñas. En muchos sitios altamente infestados no había rastros de heces de triatominos sobre la superficie de las paredes pero sí había abundantes rastros en el interior de las grandes grietas junto al armazón de madera de las paredes. Las chapas metálicas se usaban principalmente para los techos y, en muy pocos casos, para las paredes. Los domicilios con paredes únicamente de ladrillo y cemento y techo únicamente de chapa metálica representaban 36,4% de todos los domicilios; también se encontró *T. infestans* en ellos aunque con infestación (15,1%), colonización (6,2%) y disponibilidad de refugios (media ± DE = 2,9 ± 1,0) mucho menores que los domicilios con paredes de barro y/o techos de paja o chapa de cartón (32,2%, 21,6% and 3,8 ± 0,9, respectivamente).

Los depósitos y las cocinas tenían similares características de construcción a los domicilios aunque las paredes de ladrillo eran menos frecuentes (Tabla 1). Estos ecotopos frecuentemente mostraban una extraordinaria mezcla de materiales de construcción (Figura 2A,B) que refleja el cambiante acceso a los materiales a lo largo de tiempo. Los gallineros eran principalmente de madera (80,2%); < 17% de los gallineros tenía barro, ladrillos, cartón (fuese como chapa alquitranada o como cajas) o paja. Los nideros estaban construidos de manera similar a los gallineros, aunque con mayor presencia de barro y ladrillos usados principalmente para levantar pequeños tabiques que separaban los diferentes nidos (Figura 2C). Los corrales también estaban hechos mayoritariamente con madera. Los corrales de cabras tenían su cerco de troncos, tablas o alambre y rara vez incluían una pequeña porción techada (llamado 'chiquerito') para proteger a las crías; este pequeño techo era típicamente de chapa metálica o tablas de madera y, en algunos pocos casos, de chapa de cartón o paja. La infestación por *T. infestans* a nivel de sitio y el índice de disponibilidad de refugios resultaron estrecha y positivamente asociados entre sí (Figura 5). Los principales ecotopos infestados también mostraron mayores índices de disponibilidad de refugios que los demás ecotopos (Tabla 1, última columna). La concordancia entre mediciones de la disponibilidad de refugio por diferentes observadores osciló entre moderada y substancial y fue significativamente diferente de la esperada por azar (índice kappa = 0.56, z = 11.67, P < 0.0001). Las estimaciones entre diferentes observadores coincidieron en 68% de los sitios considerados y difirieron por sólo una unidad en 28% de los sitios.

Hospedadores: abundancia y distribución

Los perros y los gatos fueron los animales domésticos que más frecuentemente descansaban en los domicilios y los depósitos o cocinas, según lo informado por los moradores (Tabla S1). Los perros raramente dormían en el interior de los domicilios, cocinas o depósitos, aunque frecuentemente reposaban del lado externo junto a las paredes o en la galería. Las gallinas y otras aves de corral descansaban a la noche principalmente en los árboles y secundariamente en los gallineros. En todos los ecotopos se registraron en mayor o menor proporción pollitos. Los corrales, los baños y los hornos rara vez alojaban gallinas, perros o gatos. Las cabras, las ovejas, los cerdos y las vacas ocupaban mayormente sus respectivos corrales que tenían pocos refugios para triatominos y donde casi nunca se encontró *T. infestans*.

Etnia e infestación

En promedio, los hogares tobas constaban de familias más numerosas y jóvenes que los hogares criollos, tenían más perros y marcadamente menos aves de corral, cabras, cerdos, vacas y equinos que los criollos (Tabla 2). Las casas de tobas constaban de una menor cantidad promedio de sitios (5,8) que las de criollos (7,9), principalmente debido a menos depósitos o cocinas, corrales y sitios albergando gallinas (Tabla S2). La prevalencia de casas infestadas con *T. infestans* (según todos los métodos de captura juntos) fue 58,8% para tobas y 43,5% para criollos (χ^2 = 4,1, d.f. = 1, P = 0,04). La infestación fue mayor en los domicilios y los depósitos o cocinas de tobas y menor en los gallineros y los nideros de tobas que en los de criollos (Figura 3). La abundancia mediana de *T. infestans* fue similar entre los domicilios de ambas etnias, mucho mayor en gallineros y nideros de criollos, y sólo algo mayor en los depósitos y cocinas de tobas (debido a dos sitios con > 12 insectos capturados).

El uso de insecticidas por los moradores durante el año previo según sus propios informes fue mayor entre criollos (64%) que entre tobas (41%). Entre las familias que informaron usar insecticidas, 81,3% de los tobas y 77,5% de los criollos utilizaban aerosoles piretroides de baja concentración más de una vez al mes y 6,3% y 5,6% los utilizaban 3-12 veces/año, respectivamente. Sólo 6,3% de los tobas y 8,1% de los criollos informaron aplicar insecticidas piretroides o carbamatos de alta concentración con bombas manuales (usadas para agricultura) 1-2 veces/mes, mientras que 6,3% y 8,7% informaron aplicarlos 1-2 veces/año, respectivamente. Entre las familias que informaron usar insecticidas, la prevalencia de *T. infestans* fue menor para los criollos que para los tobas en los domicilios (19,7% vs. 28,3%), los depósitos y cocinas (11,0% vs. 18,4%) y los gallineros (9,7% vs. 14,3%), respectivamente. La prevalencia de la infestación doméstica llegó a diferir hasta 10 veces entre parajes, mientras que la

infestación peridoméstica tendió a ser menos variable entre la mayoría de los parajes (Figura 1B). De los dos parajes con mayor infestación doméstica (> 60%), uno estaba habitado sólo por familias criollas mientras que en el otro la mitad de las casas pertenecían a tobas.

Análisis multivariado

Los análisis multivariados de factores asociados con la infestación y la abundancia de *T. infestans* a nivel de sitio en los ecotopos más frecuentemente infestados se presentan en la Tabla 3. En los domicilios, depósitos y cocinas la disponibilidad de refugio mostró la mayor IR (1,00) para la infestación y la abundancia de triatominos. El uso de insecticidas por los moradores (IR = 0,99), las cantidades de personas y perros o gatos (IR = 0,95-0,96) y la presencia de techo de chapa de cartón (IR = 0,86) tuvieron mayores IR respecto a la infestación que a la abundancia de triatominos. El uso de insecticidas fue el único factor que evidenció una asociación negativa tanto con la infestación como con la abundancia. La etnia mostró una baja IR respecto a la infestación y una moderadamente alta IR respecto a la abundancia de triatominos. En los gallineros y los nideros la cantidad de gallinas fue importante para la infestación y la abundancia, mientras que la presencia de cartón, paja o barro fueron más importantes respecto a la infestación que a la abundancia, siempre con un efecto positivo. La disponibilidad de refugios no evidenció una clara importancia en el caso de los gallineros y los nideros.

El modelo logístico promediado de la infestación en domicilios, depósitos y cocinas tuvo un ajuste marginalmente significativo (Hosmer-Lemeshow χ^2 = 14,9, 8 g.l., P = 0,06). El área bajo la curva ROC fue 0,702; la sensibilidad fue pobre (49%) y la especificidad fue mayor (82%), con 76% de las observaciones correctamente clasificadas. El modelo promediado correspondiente a gallineros y nideros tuvo un buen ajuste (Hosmer-Lemeshow χ^2 = 4,5, 8 g.l., P = 0,81); el área bajo la curva ROC fue 0,759; 65% de sensibilidad y 71% de especificidad, con 70% de las observaciones correctamente clasificadas.

Los modelos binomiales negativos promediados correspondientes a ambos grupos de ecotopos mostraron correlaciones significativas entre las abundancias de triatominos observadas y las predichas (para domicilios, depósitos y cocinas, r = 0,23; para gallineros y nideros, r = 0,61). Sin embargo, estos modelos subestimaron considerablemente la frecuencia de valores nulos de abundancia y sobrestimaron los valores mayores de abundancia (datos no mostrados).

Análisis espacial

La distribución espacial de los sitios infestados por *T. infestans* (según cualquier método de captura) se analizó sólo para los ecotopos más frecuentemente infestados. Los sitios infestados mostraron una agregación significativa a distancias entre 0,8-2,5 km (Figura 6). Esta agregación se mantuvo significativa incluso tras considerar en el análisis la probabilidad de encontrar cada sitio infestado según un modelo logístico que incluía el uso de insecticidas, el ecotopo, la disponibilidad de refugios, los materiales de construcción y la cantidad de cada tipo de hospedadores.

Discusión

Valiéndonos de un abordaje de inferencia multi-modelo encontramos que la disponibilidad de refugios adecuados para *T. infestans*, el cartón como material de construcción y la cantidad de hospedadores (personas, perros, gatos y gallinas) estaban fuerte y positivamente asociados con la infestación y la abundancia de triatominos, mientras que la asociación con el uso de insecticidas por los moradores fue negativa en esta área rural de alto riesgo del Chaco argentino. Es notable que la etnia mostrara una leve o nula asociación con la infestación al considerar simultáneamente otros factores.

La disponibilidad de refugios resultó una variable clave para describir la presencia y abundancia de *T. infestans* a nivel de sitio en domicilios, depósitos y cocinas, tanto en los análisis univariados y multivariados, mostrando una mayor importancia relativa que los materiales de construcción o la cantidad de hospedadores. La disponibilidad de refugios fue también moderadamente importante para la abundancia de triatominos en gallineros y nideros. Experimentalmente ya se había demostrado que la disponibilidad de refugios es esencial para el desarrollo de colonias de *T. infestans* [20], pero la mayoría de los estudios a campo consideraron su efecto sobre la infestación doméstica sólo indirectamente a través

del tipo de materiales utilizados en paredes y techos, mientras que algunos consideraron explícitamente el grado de agrietamiento de la paredes [21,22,33]. Sin embargo, las grietas en las paredes representan sólo una fracción de los refugios posibles en sitios con paredes, tales como domicilios, depósitos y cocinas. El índice de disponibilidad de refugios probablemente capturó parte de los efectos del barro como material de construcción en la medida en que ambos factores estaban moderadamente correlacionados (r = 0,38). La utilización de barro ha sido consistentemente reconocido como un importante factor asociado con la infestación en diversos lugares del Gran Chaco y otras regiones [34] porque usualmente se agrieta (Figura S1A) y provee refugios adecuados para los triatominos, excepto cuando está bien mantenido (Figura S1G) [26]. Sin embargo, en nuestro análisis multivariado la disponibilidad de refugios, el barro y la paja resultaron moderadamente asociados con la infestación de gallineros y nideros, y sin asociación alguna con la infestación de domicilios, depósitos y cocinas. A pesar de que la estimación de la disponibilidad de refugios pueda parecer una medida subjetiva e imprecisa, la concordancia entre diferentes evaluadores experimentados y familiarizados con el área de estudio resultó de moderada a substancial. Por lo tanto, la disponibilidad de refugios podría utilizarse como un indicador de la calidad de un hábitat para *T. infestans*.

Una notable demostración del efecto de la disponibilidad de refugios sobre la infestación por *T. infestans* la aportan los corrales de cabras. Debido al tipo de construcción, la mayoría de los corrales en Pampa del Indio ofrecían poco o ningún refugio para triatominos y, de hecho, rara vez estuvieron infestados (Figura S1I,J). En cambio, los corrales de cabras en el Chaco seco tienen cercos que suelen estar hechos de ramas apiladas de arbustos espinosos y una porción techada con paja o tierra; estos corrales suelen estar altamente infestados, tanto antes como poco tiempo de después de ser rociados con piretroides [8,35].

El cartón (fuese como chapa alquitranada o cajas) usado en los nideros, gallineros y corrales resultó asociado positiva y directamente con la infestación: el cartón provee excelentes refugios para *T. infestans* a la vez que es fácil de revisar. En cambio, en los domicilios, depósitos y cocinas (donde el cartón como material de construcción está presente sólo en el techo como chapa alquitranada, Figura 2A) ningún triatomino se capturó en el cartón en sí mismo. Esto no es muy sorprendente porque los techos son más difíciles de revisar, especialmente los techos altos. Los techos de chapa de cartón también pueden estar relacionados con la infestación indirectamente ya que son más propios de hogares de menores recursos. Los pobladores rurales nos informaron que las chapas de cartón eran mucho más baratas que las de metal y más fáciles de conseguir que la paja adecuada para techos (*Spartina densiflora* o espartillo). El uso del cartón es un ejemplo de lo variables y complejas que pueden ser las relaciones entre un material de construcción tan sencillo a primera vista con otros factores relevantes.

El uso de insecticidas por los moradores mostró una alta IR en los domicilios, cocinas y depósitos. El uso frecuente de insecticidas en Pampa del Indio podría explicar, al menos en parte, por qué la prevalencia de infestación por *T. infestans* encontrada resultó mucho más baja de lo esperado para una zona como ésta, teniendo en cuenta que el último control con insecticidas tuvo lugar 12 años atrás, y que otras zonas en el Chaco seco sufrieron una rápida reinfestación después de rociados masivos [4,8,9,36]. El uso de insecticidas podría también reflejar en alguna medida el nivel económico del hogar y/o la atención y respuesta de sus moradores a la presencia de triatominos y otras alimañas [22]. Además, nuestra área de estudio presentó una relativamente alta proporción de casas de ladrillo, cemento y chapa metálica (36,4%). A pesar de que tales materiales de construcción no impiden completamente la invasión y la colonización por triatominos, muy probablemente sean menos propicios que los ranchos de barro y paja. Los pobladores locales frecuentemente evocaron que *T. infestans* solía ser mucho más abundante décadas atrás, atribuyendo su disminución a las pasadas campañas de control (tradicionalmente carentes de aspectos educativos y de continuidad con un sistema de vigilancia y control), al uso de insecticidas domésticos, al menor uso de paja en los techos y barro en las paredes, y a la deforestación. Los pobladores suelen considerar al bosque circundante como la principal fuente de *T. infestans*. A pesar de que no se hallaron focos silvestres de *T. infestans* en el área de estudio (Alvarado-Otegui *et al.*, datos no publicados), sí se encontraron en otros lugares del Gran Chaco [37-39]. Los reportes de los moradores junto con la gran heterogeneidad de materiales registrada (Figura 2B) indicaría una lenta y gradual tendencia al mejoramiento de la vivienda rural en esta zona del Chaco.

La cantidad de hospedadores de ciertas especies a nivel de sitio fue un factor importante para describir la infestación y la abundancia de *T. infestans*. Los perros, gatos y personas estuvieron positivamente asociados con la variación de la infestación en domicilios, depósitos y cocinas, al igual que en el Chaco seco [21,22] y que en otras zonas con otros triatominos [40-42]. La cantidad de gallinas (y otras aves de corral) fue muy relevante para la infestación en gallineros y nideros, como también lo fue en otras zonas del Chaco seco argentino [17,22,43]. No obstante, no se detectaron tales efectos en los domicilios, depósitos y cocinas, probablemente debido a la relativa imprecisión de los reportes de los moradores sobre la presencia de hospedadores en cada sitio y a la baja frecuencia de aves anidando en los domicilios en comparación con áreas de estudio previas.

Las diferencias en los indicadores de salud entre etnias abundan en la literatura [44] pero no pudimos encontrar comparaciones del nivel de infestación con triatominos entre criollos e indígenas dentro de una misma área. Nuestros resultados mostraron que las casas tobas, que presentaron varias diferencias demográficas y un menor uso de insecticidas que las criollas, tuvieron mayor infestación en los domicilios, depósitos y cocinas. Estas diferencias son consistentes con la mucho mayor prevalencia de *T. cruzi* en tobas (69,9%) que en criollos (40,4%) encontrada en un área cercana a la presente [45], mientras que 54% de los tobas resultó seropositivo en una zona no especificada de Pampa del Indio [46]. Sin embargo, en nuestro análisis multivariado, al considerar la etnia simultáneamente con otras variables relevantes, su relación con la infestación no resultó importante. La etnia sería relevante para entender la distribución de *T. infestans* en la medida en que implica factores directamente relacionados con la infestación (por ej., el uso de insecticidas). Enfocarse excesivamente en la etnia, concebida principalmente como aspectos culturales, puede ocultar otras diferencias relevantes entre y dentro de los tobas y los criollos. Estas diferencias podrían relacionarse con los recursos disponibles, el uso de insecticidas, los tipos y la cantidad de sitios peridomésticos, y características demográficas. De hecho, documentamos grandes diferencias en la infestación entre y dentro de los parajes estudiados, incluso entre aquellos sólo habitados por criollos. La cuestión de si las diferencias de la infestación entre criollos y tobas reflejan diferencias promedio en la posición social y económica dentro y entre estos grupos [47], en lugar de reflejar mayormente diferencias culturales o comportamentales que afectan la infestación, merece mayor estudio.

Un notable hallazgo de nuestro estudio es la marcada heterogeneidad en la distribución de la infestación, los patrones de construcción (Figura S1) y los varios factores de riesgo dentro de un área rural aparentemente homogénea. La infestación doméstica llegó a diferir 10 veces entre los parajes estudiados y hubo una agregación espacial significativa de la infestación de los sitios. Mientras algunas casas estaban altamente infestadas, 54% de las casas no evidenciaron infestación a pesar de la ausencia de acciones recientes de control vectorial. Además, la presencia de *T. infestans* se extendió por toda el área de estudio. Estos dos hallazgos implican que *T. infestans* tuvo suficiente tiempo y oportunidades para alcanzar los sitios más propicios para colonizar [4]. La agregación espacial significativa de los sitios infestados en un rango de 0,8-2,5 km sugiere la existencia de otros factores relacionados con la infestación no contemplados en nuestros modelos. Factores tales que podrían contribuir o subyacer a la presencia agregada de *T. infestans* serían: el nivel económico del hogar (por ej., afectando la calidad o el mantenimiento de la vivienda o el acceso a insecticidas), grupos familiares extendidos (que usualmente viven en casas cercanas y probablemente comparten similares tipos de vivienda y prácticas domésticas y productivas), grupos culturales (tales como los tobas, que suelen agruparse por zona) y la productividad (en tanto afecta fuertemente el nivel económico); todos estos factores bien pueden estar agregados espacialmente e interrelacionados. Esto subraya la necesidad de identificar variables socio-económicas relevantes para mejorar la comprensión de la estructura y la dinámica de este sistema.

La disponibilidad de hospedadores y las características locales de los hábitats propicios para triatominos mostraron grandes diferencias en comparación con otras área de estudio en el Chaco seco argentino [9,22,35]. El uso y la relevancia para la infestación de las chapas metálicas y de cartón alquitranado contrastan con su escasez en otras zonas de la región. Los nideros, altamente infestados y difundidos en Pampa del Indio (Figura 2C), prácticamente no existen en el Chaco seco. Los corrales de cabras rara vez albergaron *T. infestans* en Pampa del Indio, mientras que suelen estar muy infestados en el

Chaco seco por razones arriba mencionadas. Los domicilios en Pampa del Indio están mayormente techados con chapa de metal o cartón y ocasionalmente con paja (Figura S1C,G), las galerías son escasas y pequeñas (Figura 2B) y la mayoría de los triatominos se recolectaron en las paredes y los enseres. En cambio, en el Chaco seco la mayoría de los domicilios tienen techos de paja y tierra (en los que abunda *T. infestans*) y grandes galerías donde los habitantes duermen en la época de calor [21,22,48,49]. Estos patrones contrastantes probablemente surjan de diferencias en: el clima (por ej., los techos de tierra y paja se deterioran rápido con la mayor cantidad de lluvias en el Chaco húmedo); el manejo de los animales domésticos (influido por las tradiciones, las necesidades y las posibilidades locales); prácticas políticas locales (por ej., frecuentemente los candidatos políticos en Pampa del Indio entregan sin costo chapas de metal o cartón), y mayores niveles de actividad de las organizaciones de caridad. Junto con la rica diversidad ambiental, cultural y climática dentro del Gran Chaco, nuestros resultados subrayan la necesidad de considerar las especificidades y los procesos locales (contemplando también las prácticas de los moradores), en la medida en que éstas varían ampliamente dentro de la misma región y modifican la susceptibilidad a la infestación en formas imprevistas.

Los estudios transversales observacionales a campo como el nuestro tienen limitaciones y fortalezas inherentes. Las búsquedas de triatominos por el método de hora-hombre con un aerosol desalojante es el método estándar utilizado para detectar infestaciones pero tiene sensibilidad y precisión limitadas [50,51]. Su sensibilidad para detectar infestaciones domésticas de *T. infestans* rondó 70-77% en el Chaco seco [52] pero probablemente sea menor a bajas densidades del vector. A expensas de un mayor costo, se podrían realizar repetidas búsquedas en los mismos sitios a fin de estimar la probabilidad de detección de infestación, que luego puede incorporarse en el modelado estadístico [53]. La información sobre la presencia y la abundancia de hospedadores animales y sobre el uso de insecticidas (ambos variables estacionalmente) basada en los informes de los moradores está sujeta a sesgos en cuanto a lo que se recuerda e informa y, por lo tanto, resulta algo imprecisa. Estas cuestiones probablemente subyacen el limitado ajuste de los modelos logísticos y binomiales negativos. Los análisis multivariados sólo pueden mostrar que la variabilidad registrada en las variables explicativas no tiene una relación suficientemente fuerte con la variabilidad registrada en la variable de respuesta (infestación o abundancia de triatominos, en nuestro caso). Un resultado no significativo estadísticamente puede estar reflejando que tales variables explicativas y de respuesta están genuinamente no correlacionadas o que no hay suficiente variabilidad registrada en ellas como para detectar correlación alguna. Respecto a las fortalezas del presente trabajo, cabe mencionar la detallada información recabada a nivel de sitio en un considerable número de casas en un área rural bien definida y analizada por medio de un abordaje de inferencia multi-modelo que reduce el sobreajuste.

El mejoramiento de la vivienda frecuentemente se propone como la verdadera solución para la infestación doméstica [1] pero en la práctica el rociado con insecticidas residuales continúa siendo la única herramienta utilizada para controlar la infestación de las casas por triatominos. Nuestros resultados tienen implicancias en ambas direcciones: i) El mejoramiento de la vivienda (y también de los sitios peridomésticos relevantes) tendría que promoverse más ampliamente y llevarse a cabo más efectivamente a fin de reducir la disponibilidad de refugios y así la infestación, en lugar de concentrarse sólo o principalmente en reemplazar ciertos materiales de construcción (por ej., la paja por chapas metálicas). Una casa de tipo urbano, con paredes de ladrillo y cemento y techo de chapa metálica, no garantiza la ausencia de triatominos, como se muestra en la Figura S1B,H, ya que pueden existir refugios adecuados en paredes, camas, muebles y otros enseres. A su vez, las casas de barro pueden mantenerse libres de *T. infestans* si reciben el mantenimiento adecuado (Figura S1G), se minimiza la presencia de hospedadores en los domicilios y se emplean insecticidas cuando son requeridos [22,26]. Algunas modificaciones simples y económicas de los ecotopos peridomésticos clave, tales como reducir la disponibilidad de refugios en los nideros o renovarlos más frecuentemente, pueden reducir fuertemente las poblaciones peridomésticas de *T. infestans*; ii) Mejores viviendas, con menos refugios para triatominos, podrían reducir la probabilidad de tener focos residuales luego del rociado con insecticidas [8,33] y facilitar la vigilancia vectorial comunitaria, y iii) Las heterogeneidades locales y regionales en la distribución de la infestación y los factores relevantes (incluyendo las prácticas y características socio-económicas de los

habitantes) tienen que tomarse en cuenta para priorizar y planificar las operaciones de rociado y la vigilancia vectorial más eficientemente. La preferencia de *T. infestans* por determinados hospedadores y tipos de refugios es estrictamente biológica pero la ocurrencia de estos hospedadores y refugios en una casa es también un fenómeno social que necesita abordarse más ampliamente. Para alcanzar un control vectorial y de la enfermedad efectivo y sostenible se necesita una perspectiva más integral que considere simultáneamente los procesos sociales, económicos y biológicos a nivel local y regional, junto con las posibilidades, necesidades y expectativas de la población local [47,54-56].

Referencias

1. World Health Organization (2007) Reporte sobre la enfermedad de Chagas. TDR/SWG/09.
2. Briceño-León R, Méndez Galván J (2007) The social determinants of Chagas disease and the transformations of Latin America. Mem Inst Oswaldo Cruz 102: 109-112.
3. Panamerican Health Organization (2006) XV Reunión de la Comisión Intergubernamental del Cono Sur para la Eliminación de *Triatoma infestans* y la Interrupción de la Transmisión de Tripanosomiasis Transfusional (INCOSUR-Chagas) (Brasilia, Brasil, 6-9 junio 2006). http://www.paho.org/spanish/ad/dpc/cd/dch-incosur-xv.htm.
4. Gürtler RE, Kitron U, Cecere MC, Segura EL, Cohen JE (2007) Sustainable vector control and management of Chagas disease in the Gran Chaco, Argentina. Proc Natl Acad Sci U S A 104: 16194-16199.
5. Schofield CF, Jannin J, Salvatella R (2006) The future of Chagas disease control. Trends Parasitol 22: 583-588.
6. The Nature Conservancy, Fundación Vida Silvestre Argentina, Fundación para el Desarrollo Sustentable del Chaco, Wildlife Conservation Society Bolivia (2005) Evaluación Ecorregional del Gran Chaco Americano / Gran Chaco Americano Ecoregional Assessment. Buenos Aires: Fundación Vida Silvestre Argentina.
7. Gürtler RE (2007) Eco-epidemiología regional de la transmisión vectorial: enfermedad de Chagas en el Gran Chaco. In: Silveira AC, editor. La enfermedad de Chagas a la puerta de los 100 años del conocimiento de una endemia americana ancestral. Buenos Aires: Panamerican Health Organization – Fundación Mundo Sano. OPS/CD/426-06.2007.
8. Gürtler RE, Canale DM, Spillmann C, Stariolo R, Salomon OD, et al. (2004) Effectiveness of residual spraying of peridomestic ecotopes with deltamethrin and permethrin on *Triatoma infestans* in rural western Argentina: a district-wide randomized trial. Bull World Health Organ 82: 196-205.
9. Cecere MC, Vazquez-Prokopec GM, Ceballos LA, Gurevitz JM, Zarate JE, et al. (2006) Comparative trial of effectiveness of pyrethroid insecticides against peridomestic populations of *Triatoma infestans* in northwestern Argentina. J Med Entomol 43: 902-909.
10. Andrade ALSS, Zicker F, de Oliveira RM, da Silva IG, Silva SA, et al. (1995) Evaluation of risk factors for house infestation by *Triatoma infestans* in Brazil. Am J Trop Med Hyg 53: 443-447.
11. Aguilar V H, Abad-Franch F, Racines V J, Paucar C A (1999) Epidemiology of Chagas disease in Ecuador. A brief review. Mem Inst Oswaldo Cruz 94: 387-393.
12. Cecere MC, Gürtler RE, Canale D, Chuit R, Cohen JE (1997) The role of the peridomiciliary area in the elimination of *Triatoma infestans* from rural Argentine communities. Rev Pan Salud Publica 1: 273-279.
13. Starr MD, Rojas JC, Zeledón R, Hird DW, Carpenter TE (1991) Chagas´disease: risk factors for house infestation by *Triatoma dimidiata*, the mayor vector of *Trypanosoma cruzi* in Costa Rica. Am J Epidemiol 133: 740-747.
14. Cuba Cuba CA, Abad-Franch F, Roldán Rodríguez J, Vargas Vásquez F, Pollack Velásquez L, et al. (2002) The triatomines of northern Peru, with emphasis on the ecology and infection by trypanosomes of *Rhodnius ecuadoriensis* (Triatominae). Mem Inst Oswaldo Cruz 97: 175-183.
15. Levy MZ, Bowman NM, Kawai V, Waller LA, Cornejo del Carpio JG, et al. (2006) Periurban *Trypanosoma cruzi*-infected *Triatoma infestans*, Arequipa, Peru. Emerg Infect Dis 12: 1345-1352.

16. Abad-Franch F, Monteiro FA, Jaramillo O (2009) Ecology, evolution, and the long-term surveillance of vector-borne Chagas disease: A multi-scale appraisal of the tribe Rhodniini (Triatominae). Acta Trop 110: 159-177.
17. Cecere MC, Gürtler RE, Chuit R, Cohen JE (1997) Effects of chickens on the prevalence of infestation and population density of *Triatoma infestans* in rural houses of north- west Argentina. Med Vet Entomol 11: 383-388.
18. Schofield CJ, Marsden PD (1982) The effect of wall plaster on a domestic population of *Triatoma infestans*. Bull Pan Am Health Organ 16: 356-360.
19. Schofield CJ, Briceño-León R, Kolstrup N, Webb DJT, White GB (1991) The role of house design in limiting vector-borne disease. In: Curtis CF, editor. Appropriate Technology in Vector Control. Boca Raton: CRC Press.
20. Cecere MC, Canale DM, Gürtler RE (2003) Effects of refuge availability on the population dynamics of *Triatoma infestans* in central Argentina. J Appl Ecol 40: 742-756.
21. Gürtler RE, Cecere MC, Rubel DN, Schweigmann NJ (1992) Determinants of the domiciliary density of *Triatoma infestans*, vector of Chagas' disease. Med Vet Entomol 6: 75-83.
22. Cecere MC, Gürtler RE, Chuit R, Cohen JE (1998) Factors limiting the domestic density of *Triatoma infestans* in north-west Argentina: a longitudinal study. Bull World Health Org 76: 373-384.
23. Burnham KP, Anderson DR (2002) Model selection and multimodel inference: a practical information-theoretic approach. New York: Springer-Verlag.
24. Marsden P, Virgens D, Magalhaes I, Tavares-Neto J, Ferreira RA, et al. (1982) Ecologia domestica do *Triatoma infestans* em Mambaí, Goiás-Brasil. Rev Inst Med Trop 24: 364-373.
25. Valeggia CR, Tola F (2004) Argentina Toba. In: Ember C. R. EM, editor. Encyclopedia of medical anthropology: health and illness in the world's cultures. New York: Kluwer Academic/Plenum Publishers.
26. Cecere MC, Gürtler RE, Canale D, Chuit R, Cohen JE (2002) Effects of partial housing improvement and insecticide spraying on the reinfestation dynamics of *Triatoma infestans* in rural northwestern Argentina. Acta Trop 84: 1-16.
27. Belsley DA, Kuh E, Welsch RE (2004) Regression Diagnostics: Identifying Influential Data and Sources of Collinearity. New York: John Wiley & Sons.
28. R Development Core Team (2008) R: A language and environment for statistical computing. Vienna: R Foundation for Statistical Computing.
29. StataCorp. (2005) Stata Statistical Software: Release 9.0. College Station: Stata Corp.
30. Wiegand T, Moloney KA (2004) Rings, circles, and null-models for point pattern analysis in ecology. Oikos 104: 209-229.
31. Stoyan D, Stoyan, H. (1994) Fractals, Random Shapes and Point Fields. Methods of geometrical statistics. New York: John Wiley & Sons.
32. Landis JR, Koch GG (1977) The measurement of observer agreement for categorical data. Biometrics 33: 159-174.
33. Gürtler RE, Petersen RM, Cecere MC, Schweigmann NJ, Chuit R, et al. (1994) Chagas disease in north-west Argentina: risk of domestic reinfestation by *Triatoma infestans* after a single community- wide application of deltamethrin. Trans R Soc Trop Med Hyg 88: 27-30.
34. Mott KE, Muniz TM, Lehman JSJ, Hoff R, Morrow RHJ, et al. (1978) House construction, triatomine distribution, and household distribution of seroreactivity to *Trypanosoma cruzi* in a rural community in northeast Brazil. Am J Trop Med Hyg 27: 1116-1122.
35. Ceballos LA, Vazquez-Prokopec GM, Cecere MC, Marcet PL, Gürtler RE (2005) Feeding rates, nutritional status and flight dispersal potential of peridomestic populations of *Triatoma infestans* in rural northwestern Argentina. Acta Trop 95: 149-159.
36. Porcasi X, Catalá SS, Hrellac H, Scavuzzo MC, Gorla DE (2006) Infestation of rural houses by *Triatoma infestans* (Hemíptera: Reduviidae) in a southern area of the Gran Chaco in Argentina. J Med Entomol 43: 1060-1067.

37. Ceballos LA, Piccinali RV, Berkunsky I, Kitron U, Gürtler RE (2009) First finding of melanic sylvatic *Triatoma infestans* (Hemiptera: Reduviidae) colonies in the Argentine Chaco. J Med Entomol 46: 1195-1202.
38. Rolón M, Vega MC, Román F, Gómez A, de Arias AR (2011) First report of colonies of sylvatic *Triatoma infestans* (Hemiptera: Reduviidae) in the Paraguayan Chaco, using a trained dog. PLoS Negl Trop Dis 5: e1026.
39. Noireau F, Flores R, Gutierrez T, Abad-Franch F, Flores E, et al. (2000) Natural ecotopes of *Triatoma infestans* dark morph and other sylvatic triatomines in the Bolivian Chaco. Trans R Soc Trop Med Hyg 94: 23-27.
40. Piesman J, Sherlock IA, Christensen HA (1983) Host availability limits population density of *Panstrongylus megistus*. Am J Trop Med Hyg 32: 1445-1450.
41. Ramsey J, Alvear A, Ordoñez R, Muñoz G, García A, et al. (2005) Risk factors associated with house infestation by the Chagas disease vector *Triatoma pallidipennis* in Cuernavaca metropolitan area, Mexico. Med Vet Entomol 19: 219-228.
42. Campbell-Lendrum DH, Angulo VM, Esteban L, Tarazona Z, Parra GJ, et al. (2007) House-level risk factors for triatomine infestation in Colombia. Int J Epidemiol 36: 866-872.
43. López A, Crocco L, Morales G, Catalá S (1999) Feeding frequency and nutritional status of peridomestic populations of *Triatoma infestans* from Argentina. Acta Trop 73: 275-281.
44. Montenegro RA, Stephens C (2006) Indigenous health in Latin America and the Caribbean. Lancet 367: 1859-1869.
45. Biancardi MA, Moreno MC, Torres N, Pepe C, Altcheh J, et al. (2003) Seroprevalencia de la enfermedad de Chagas en 17 parajes del “Monte Impenetrable” de la Provincia del Chaco. Medicina (B Aires) 63: 125-129.
46. Alonso J, Fabre A, Galván M, Lucero R, Brusés B, et al. (2009) La enfermedad de Chagas en poblaciones aborígenes del noreste de Argentina. Enf Emerg 11: 115-118.
47. Farmer P (2001) Infections and Inequalities: the Modern Plagues. Berkeley: University of California Press.
48. Urquijo H (1969) Tipos predominantes de vivienda natural en la República Argentina, Vr.2/IIV.4. Buenos Aires: Instituto Nacional de Colonización y Régimen de la Tierra, Instituto de Investigaciones de la Vivienda. Facultad de Arquitectura y Urbanismo, Universidad de Buenos Aires.
49. Ronderos RA, Schnack JA, Ghilini JM, Spinelli GR (1981) Estudio ecológico sobre una población domiciliaria de *Triatoma infestans* Klug de la provincia biogeográfica chaqueña. Ecosur 8: 1-24.
50. Schofield CJ (1978) A comparison of sampling techniques for domestic populations of Triatominae. Trans R Soc Trop Med Hyg 72: 449-455.
51. Pinchin R, Fanara DM, Castleton CW, Oliveira Filho AM (1981) Comparison of techniques for detection of domestic infestations with *Triatoma infestans* in Brazil. Trans R Soc Trop Med Hyg 75: 691-694.
52. Gürtler RE, Chuit R, Cecere MC, Castañera MB (1995) Detecting domestic vectors of Chagas disease: a comparative trial of six methods in north-west Argentina. Bull World Health Organ 73: 487-494.
53. Abad-Franch F, Ferraz G, Campos C, Palomeque FS, Grijalva MJ, et al. (2010) Modeling disease vector occurrence when detection is imperfect: infestation of Amazonian palm trees by triatomine bugs at three spatial scales. PLoS Negl Trop Dis 4: e620.
54. Monroy C, Bustamante DM, Pineda S, Rodas A, Castro X, et al. (2009) House improvements and community participation in the control of *Triatoma dimidiata* re-infestation in Jutiapa, Guatemala. Cad Saude Publica 25: S168-S178.
55. Breilh J (2003) Epidemiología crítica: Ciencia emancipadora e interculturalidad. Buenos Aires: Lugar Editorial.
56. Lebel J (2003) Health: an ecosystem approach. Ottawa: Stylus Pub Llc.

Figura 1. Distribución de la infestación por *T. infestans* a nivel de casa según capturas por método de hora-hombre en Pampa del Indio.
A: Mapa del área de estudio con las casas habitadas, los parajes y el estado de infestación. B: Prevalencia doméstica y peridómestica de infestación según el paraje; los diamantes representan el porcentaje de casas habitadas por tobas en cada paraje; los números sobre las barras indican la cantidad de casas habitadas en cada paraje; las líneas verticales indican ± 1 desviación estándar. Abreviatura de parajes: 10M, 10 de Mayo; CT, Campo Los Toros; CO, Colonia Ombú; SV-CV, El Salvaje- Los Ciervos; FB, Fortín Brown; H, La Herradura; LL, La Loma; BV, Las Bravas; CHU, Las Chuñas; RI, Santa Rita; LUG, Santos Lugares, and 3L, Tres Lagunas.

Figura 2. Estructuras típicas de la zona rural en Pampa del Indio.
A: Domicilio y parte de peridomicilio mostrando una variedad de materiales en los techos (chapas metálica, chapas de cartón alquitranado y paja). B: Un domicilio, en parte con paredes de barro y palo a pique, en parte con ladrillos cocidos asentados sobre barro, y techo de chapa metálica. C: Un nidero de barro, donde las gallinas y otras aves de corral anidan.

Figura 3. Prevalencia de la infestación y abundancia mediana de *T. infestans* según ecotopo y etnia.
Los datos corresponden a capturas por método de hora-hombre. Los números sobre las barras indican la cantidad de sitios evaluados por infestación en cada categoría. Las líneas verticales indican ± 1 desviación estándar.

Figura 4. Abundancia de *T. infestans* por sitio según ecotopo.
Abundancia determinada por método de hora-hombre. Símbolos gris claro, abundancia mediana; cajas, rango intercuartil; líneas verticales, valores adyacentes al rango intercuartil; símbolos oscuros, valores extremos.

Figura 5. Prevalencia de la infestación por *T. infestans* según la disponibilidad de refugios en los ecotopos mayormente infestados.
Los números sobre los puntos indican la cantidad de sitios evaluados en cada categoría. Las líneas verticales indican ± 1 desviación estándar.

Figura 6. Análisis espacial de la infestación por *T. infestans* en los sitios mayormente infestados.
Sólo se consideran los domicilios, cocinas, depósitos, gallineros y nideros. C.E.: rango de confianza (‘confidence envelope’) según el modelo nulo de marcas al azar bajo un modelo homogéneo de Poisson.

Tabla 1. Uso de materiales de construcción y disponibilidad de refugios (media ± desviación estándar, SD) según ecotopo.

Tabla 2. Hospedadores domésticos de cada tipo por casas habitada según etnia.

Tabla 3. Importancia relativa y efectos de las variables respecto a la infestación y la abundancia de *T. infestans* a nivel de sitio.
